# Humic Acid Alleviates Fe Chlorosis in Graminaceous Plants Through Coordinated Fe-Dependent and Fe-Independent Mechanisms

**DOI:** 10.3389/fpls.2022.803013

**Published:** 2022-02-02

**Authors:** Maria Garnica, Roberto Baigorri, Sara San Francisco, Angel M. Zamarreño, Jose M. Garcia-Mina

**Affiliations:** ^1^BACh Research Group, Department of Environmental Biology, Instituto de Biodiversidad y Medioambiente (BIOMA), University of Navarra, Pamplona, Spain; ^2^Centre Mondial de l’Innovation (CMI) – Groupe Roullier, Saint-Maló, France

**Keywords:** humic, graminaceous, iron chlorosis, phytosiderophore, cytokinins, plant growth promotion, wheat

## Abstract

Many studies have shown the close relationship between the beneficial action of soil and sedimentary humic acids on the growth of plants cultivated in calcareous soils and their ability to improve Fe plant nutrition. These results have been ascribed to the humic acid (HA) capability to improve Fe solubility and bioavailability. However, other effects more related to a humic acid action on the specific mechanisms activated in roots of plants under Fe deficiency cannot be ruled out. Although this question has been studied in dicotyledonous plants, in graminaceous plants there are no specific studies. Here we investigate the ability of a humic acid extracted from peat (HA) to improve Fe nutrition in wheat plants cultivated under Fe deficient and sufficient conditions. The results show that HA can improve the physiological status of Fe deficient wheat plants by alleviating some of the deleterious consequences of Fe deficiency on plant development and increasing the plant ability to secrete phytosiderophores to the nutrient solution. This action of HA is associated with increases in the Fe-active pool in leaves that might be related to the mobilization of the Fe complexed by HA resulting from the interaction of HA with the phytosiderophores in the nutrient solution. The Fe translocation from the root to the shoot may be favored by the action of *trans*-Zeatin Riboside (tZR) since the leaf concentration of this phytohormone was enhanced by HA in Fe deficient plants.

## Introduction

Iron (Fe) is a key element for energetic processes in plants, such as photosynthesis, respiration, nitrogen assimilation, and synthesis of plant enzymes (Barton and Abadia, 2006; Abadía et al., 2011). In the soil, the content of Fe is higher than that of any other microelement; however, its bioavailability is highly dependent on changes in the acidity and redox potential of the environment (Lucena, 2003). This fact favors its solubility in acidic soils. However, in calcareous soils it forms highly insoluble compounds, such as hydroxides-oxides and carbonates, which make it unavailable to plants (Lucena, 2003).

Hence, Fe deficiency is one of the major nutritional concerns lowering crop yield and nutritional quality, mainly in alkaline-calcareous soils, making up 30% of the world’s agricultural soils (Barton and Abadia, 2006; Abadía et al., 2011). Nonetheless, the levels of Fe in soil solution can increase due to complexation of the micronutrient with soluble organic ligands like organic acids, phenols, phytosiderophores released by roots of graminaceous plants, microbial siderophores, and humified organic matter (Garcia-Mina, 2006; Zanin et al., 2019; Gerke, 2021).

Humic substances (HS) are principal components of soil organic matter and dissolved organic matter (Chen et al., 2004a; Rose et al., 2014; Olaetxea et al., 2018; Nardi et al., 2021). The presence of HS is a significant factor influencing soil fertility (Chen et al., 2004a; Olaetxea et al., 2018). These molecules have ecological importance, as they intervene in regulating a large number of chemical and biological processes that occur in natural ecosystems (Chen et al., 2004a; Olaetxea et al., 2018; Nardi et al., 2021). Their ability to improve plant growth has been well-established in diverse plant species and growth conditions, although the mechanism responsible for this biological action is poorly understood. Many studies have demonstrated that HS favor the growth and development of plants by facilitating the input of different elements, principally Fe copper (Cu) and zinc (Zn) (Chen et al., 2004a,b; Garcia-Mina et al., 2004; Zanin et al., 2019; Gerke, 2021). Moreover, HS also promotes plant growth through action on plant metabolism and physiology, which derives from the interaction of HS with plant roots (Canellas et al., 2015; Olaetxea et al., 2015, 2018, 2019; Nardi et al., 2021).

As far as Fe nutrition is concerned, humified organic matter may provide a reservoir of this micronutrient that plant root exudates can utilize (Zanin et al., 2019; Gerke, 2021) due to their chelating activity (Chen et al., 2004a,b; Garcia-Mina, 2006; Zanin et al., 2019; Gerke, 2021). Several studies have shown the ability of humic complexes with different metals, including Fe, Cu, or Zn, to provide available micronutrients to diverse plant species cultivated under different conditions and soil types (Garcia-Mina et al., 2004; Cieschi et al., 2017; Cieschi and Lucena, 2018).

Besides the positive impact of HS complexes enhancing the iron input to plants, the presence of HS in the nutrient solution also has beneficial effects on the physiology and metabolism of plants suffering Fe deficiency (Olaetxea et al., 2018; Zanin et al., 2019). Abros’kin et al. (2016) reported that the treatment with a sedimentary humic acid improved the antioxidant status and activated the lipid metabolism of plants growing under Fe deficiency. In this line, several studies have reported the ability of HS to enhance the specific root responses to Fe deficiency in dicotyledonous plants (Aguirre et al., 2009; Zanin et al., 2019). Fe-HS complexes increased the activity of H + -ATPase acidifying the rhizosphere, nitrate root uptake rates (Varanini et al., 1993; Pinton et al., 1999; Nardi et al., 2002; Quaggiotti et al., 2004), and Fe(III)-chelate reductase activity at the root surface (Pinton et al., 1997), in Fe-deficient cucumber plants. In this line, several studies have shown that Fe-HS complexes can induce the up-regulation of the central genes involved in Fe-deficiency response (FRO1, IRT1, and IRT2) in tomato (Tomasi et al., 2013). Aguirre et al. (2009) reported these effects of HS at both molecular and enzymatic levels, even in Fe-sufficient cucumber plants.

However, the studies on the effect of HS on graminaceous plants cultivated under Fe limiting conditions are scarce. Likewise, there is no information about the possible effects of HS on the specific response of this type of plants to Fe deficiency, principally the biosynthesis of phytosiderophores and their release from the root to the rhizosphere (Garnica et al., 2018; Zanin et al., 2019; Gerke, 2021).

According to Chen et al. (2004b), the only presence of HS in the nutrient solution of wheat plants grown under iron deficiency conditions did not significantly enhance chlorophyll biosynthesis. It was needed the presence of Fe and Zn salts, which formed organo-metal complexes with HS, to increase chlorophyll levels and improve plant growth. However, Abros’kin et al. (2016) observed that highly purified (almost Fe free) HS enhanced the electron transport rate (ETR), sterols content, and the growth of Fe-deficient wheat plants cultivated in hydroponics.

Thus, HS have beneficial effects on graminaceus plants growing under Fe limited conditions in hydroponics. However, it is not clear whether this beneficial effect is due to the general physiological effects usually associated with HS or to specific actions of HS on the mechanisms involved in the plant response to Fe deficiency. Likewise, the role of the Fe constitutively complexed in HA main structure in all these effects remains unclear.

Recently, some studies have reported that auxin has a key role in controlling phytosiderophores’ root release of wheat plants subjected to Fe deficiency (Garnica et al., 2018). On the other hand, many studies have revealed the central role of auxin (mainly indolacetic acid and IAA) in the whole effects of HS on root functionality in both dicotyledonous and graminaceous plants (Canellas and Olivares, 2014; Olaetxea et al., 2018; Nardi et al., 2021). This role of auxin was coordinated with effects on other phytohormones, such as ethylene, nitric oxide (NO), and abscisic acid (ABA) (Mora et al., 2012, 2014; Olaetxea et al., 2015, 2018). Recently, Divte et al. (2019) reported the role of ethylene in the regulation of Fe-deficiency root responses in different wheat varieties. In this context, we hypothesize that HS might affect the specific responses of grasses to Fe deficiency due to changes in the Fe distribution within the plant and the concentration and activity of some phytohormones in roots and shoots. These HS-mediated effects might involve Fe-dependent (the role of Fe complexed in HS) and Fe-independent mechanisms (direct effect of the organic moiety of HS).

In order to investigate this hypothesis, the effects of a humic acid extracted from peat (HA) on the growth traits, iron plant distribution, the concentration of several phytohormones [ABA, IAA, and cytokinins (CKs)] in plant tissues, and phytosiderophores root release, of wheat plants cultivated in the presence of different levels of Fe in the nutrient solution have been studied. The study included a control receiving the amount of Fe contained in HA composition after its extraction and purification.

## Materials and Methods

### Extraction, Purification, and Characterization of the Sedimentary Humic Acid

The humic acid (HA) employed in the experiments was extracted and purified from a peat-based substrate as described in Jannin et al. (2012).

Humic acid was extensively characterized by several complementary analytical techniques (Jannin et al., 2012; Supplementary Figures S1.1, S1.2 and Supplementary Tables S1.1, S1.2).

Humic acid contained 39.65% of C and 0.69% of Fe. The concentration of the other elements that were detected in HA were 0.01% Cu and 0.0012% Zn.

### Plant Culture, Experimental Design, and Mineral Analysis

Seeds of *Triticum aestivum* (cv. *Bermude*) were germinated with distilled water in 300 mL plastic opaque pots containing perlite, in a germinating chamber in the darkness and at a temperature of 25^°^C and 85% relative humidity for 10 days. After germination, the seedlings were grouped into two groups, one receiving Fe and the other without Fe, and transferred to 15 L pots and grown in aerated hydroponic culture for 15 days in a growth chamber. Fe-sufficient plants were grown with 89 μM EDTA-Fe, while Fe-deficient plants were grown without Fe to induce iron deficiency. The nutrient solution containing the other nutrients were the same for all plants and contained 2 mM KNO_3_, 0.5 mM Ca(NO_3_)_2_, 1 mM Mg(NO_3_)_2_, 1 mM K_2_SO_4_, 1 mM KH_2_PO_4_, 2 mM KNO_3_, 0.5 mM Ca(NO_3_)_2_, 1 mM KH_2_PO_4_, 1 mM K_2_SO_4_, 18 μM EDTA-Mn, 0.9 μM EDTA-Cu, 1.75 μM EDTA-Zn. The nutrient solutions were renewed every week. The initial pH of the nutrient solution was 6.0 and did not show significant changes during the experiment. The growth chamber conditions were as follows: 24/18°C and 50–70% relative humidity with a 15/9 h day/night photoperiod (irradiance: 250 μmol m^–2^ s^–1^ photosynthetically active radiation).

After 15 days, the plants belonging to the two groups (+Fe and –Fe) were transferred to renewed nutrient solutions, and the following treatments were applied depending on the experiment:

#### First Experiment

The treatments included plants growing with Fe (+Fe); plants growing without Fe (–Fe), plants growing with Fe plus HA (100 mg C⋅L^–1^) (+Fe+HA), and plants growing without Fe plus HA (100 mg C L^–1^) (Fe + HA).

#### Second Experiment

The treatments included plants growing with Fe (+Fe), plants growing without Fe (–Fe); plants growing without Fe plus HA (100 mg C⋅L^–1^); and plants growing with the amount of Fe provided by HA-structure (+12.5 μM Fe).

The concentration of HA applied to the nutrient solution is expressed as C and is equivalent to 252.21 mg L^–1^ of HA (the content of C in HA is 39.65%).

Each treatment consisted of three replicates with 23 plants per replicate (69 plants in total). The two experiments were not combined in only one experiment due to the high number of plants involved, making it difficult to manage the experiment correctly.

Each experiment was replicated twice.

Plants were harvested 4, 72, and 96 h after treatment application. The harvests were conducted at the same time of the day to exclude diurnal variations, which meant 8 h after the start of the light period. Shoots and roots were dried at 60^°^C for dry matter evaluation and the analysis of total Fe and the Fe fraction soluble in 0.1 M HCl. The total Fe and a the concentration of the main mineral nutrients were determined after acidic digestion of dried samples. Soluble Fe was determined after extraction of fresh samples in 0.1 M HCl (1:10) for 16 h at room temperature. Analyses were carried out by ICP-OES.

A specific portion of the shoots and roots was quick-frozen in liquid nitrogen for hormone and gene expression analyses.

### Phytosiderophores Determination

Root exudates were collected for the measurement of phytosiderophore release from roots at each harvest time. For the collection of root exudates, intact plants were removed from the nutrient solution and the roots were washed with deionized water. After that, plants were placed in 200 mL aerated distilled water for 3 h. Root exudates were collected and frozen at −40^°^C following previous treatment with Micropur (Katadyn, Lindau, Switzerland) to prevent microbial degradation. The study used three replicates (23 plants per replicate) per treatment and harvest time.

Phytosiderophores were measured as described in Reichman and Parker (2007). Briefly, a 10 mL aliquot of sample solution was dispensed into a vial and 10 mL of a blank of deionized water was dispensed into a separate vial. 0.5 mL of 0.6 mM FeCl_3_ was added to each vial. All vials were shaken for 15 min, and then 1 mL of 1.0 M Na-acetate buffer (pH 7.0) was added and the solutions were shaken for 10 min. To reduce Fe (III) to Fe (II), the solutions were filtered through a 0.2 μm filter into 0.25 mL of 6 M HCl, and then 0.5 mL of 80 g⋅L^–1^ hydroxylamine hydrochloride was added. All the solutions were placed in an oven at 50–60°C for 30 min. After incubation, 0.25 mL of 2.5 g⋅L^–1^ Ferrozine and 1 mL of 2.0 M Na-acetate buffer (pH 4.7) were added to the solution. Finally, the tubes were shaken briefly to mix the contents, and after 5 min, the absorbance at 562 nm was determined. The absorbance readings were converted into concentration using the Beer-Lambert law against a reference curve prepared with adequate Fe standards. It was assumed that the stoichiometry of reaction is 1:1.

### Determination of Chlorophyll Concentration and Net Photosynthetic Rates

During the experiment, determinations of chlorophyll concentration and net photosynthetic rates were monitored before each harvest time. The relative chlorophyll concentration was determined using a non-destructive method: a Dualex chlorophyll meter (Force-A Dualex Scientific, Orsay, France). The determination was carried on ten measures per leaf and five leaves per replicate. Moreover, the net photosynthetic rate was measured using a CIRAS-3 photosynthesis system (PPSystems, Amesbury, MA, United States).

### Nitrate Extraction and Quantification

Shoot- and root- NO_3_ concentrations were analyzed from an aqueous extraction performed at 80^°^C for 1 min and ground with an ultraturrax. Extraction was filtered through filter paper and syringe filter of 0.45 μm. NO_3_ was determined by ion-exchange chromatography (Dionex Corporation, Sunnyvale, CA, United States).

### Total Nitrogen Content Analysis

Plant N content was analyzed in dried samples of roots and shoots. N concentration was determined using a LECO CHN-2000 elemental analyzer (LECO Corporation, St. Joseph, MI, United States).

### Extraction and Quantification of IAA and Abscisic Acid in Plants Tissues

The extraction and purification of IAA and ABA and its quantification were carried out according to the method described in Bacaicoa et al. (2011).

### Cytokinin Extraction and Purification

Endogenous cytokinins were extracted and purified following the procedure described in Garnica et al. (2010). The CKs quantified were: *trans*-Zeatin Riboside (tZR), *cis*-Zeatin Riboside (cZR), isopentyladenine (iP), and isopentyladenosine (iPR).

### Real-Time Quantitative RT-PCR Analysis

The roots of the plants were collected and ground to a powder with liquid nitrogen prior to RNA extraction. Total RNA was extracted from between 50 and 90 mg of crushed root using a mix of 350 μL of guanidinium-thiocyanate lysis buffer and 3.5 μL of β-mercaptoethanol of NucleoSpin RNA Plant Kit (Macherey-Nagel, Düren, Germany). Following this, treatment of RNA with DNase was performed according to the manufacturer‘s recommendations. After washing extracted RNA with dry silica membranes provided by the kit, RNA purity and concentration was quantified by fluorescence-based Experion RNA STdSens Analysis kit (Biorad). First-strand cDNA synthesis was carried out in 20 μL reactions containing 1 μg of RNA with RNase H^+^ MMLV reverse transcriptase iScript and a mix of oligo(dT) and random hexamer primers from iScript cDNA Synthesis Kit (Bio-Rad Laboratories, Hercules, CA, United States). The reverse transcription was made up for 5 min at 25°C, 30 min at 42°C, and ended by 5 min at 85°C. The gene expression was analyzed with the CFX384 Touch Real-Time qPCR detection System (Bio-Rad Laboratories) using iQ SYBR Green supermix (Bio-Rad) containing hot-start iTaq DNA polymerase in 10 μL reaction volume with 1 μL of cDNA.

Primer pairs used in PCR amplification for each gene studied

**Table:** 

Gene name	Gene code	Fwd sequence (5′→ 3′)	Rev sequence (5′→3′)	PCR conditions
RLIa	XM_044519073	TTGAGCAA CTCATGGA CCAG	GCTTTCCAAG GCACAAACAT	40× (10 s 95^°^C, 10 s 55^°^C, 20 s 72^°^C)
NAAT	XM_044594834	TCATCATA AACCCAAA CAATCC	TATACCT CGTCAGCAA TCAC	40× (10 s 95^°^C, 10 s 52.3^°^C, 20 s 72^°^C)
RUBISCO	XM_044518499	CGTGATG GTCGTATG GAGAAG	ACATTGT CGGTCTGGAA GATAC	40× (10 s 95^°^C, 10 s 54.9^°^C, 20 s 72^°^C)
NT 1.2	XM_044520025	CGGTATT CACAGCCA GAGGAG	GCCCAG CCATCATC AGGTAG	40× (10 s 95^°^C, 10 s 60^°^C, 20 s 72^°^C)
NT 2.1	XM_044552779	CACTTCC ACCTTGAC CTTC	AGAGAGA TAGCCACCC ATAG	40× (10 s 95^°^C, 10 s 56^°^C, 20 s 72^°^C)
SULTR 2.1	XM_044506503	CCGGATC TCTATCCTC GTGCTA	GATGAAA GTCGCGTTG ATGAAGC	40× (10 s 95^°^C, 10 s 63^°^C, 20 s 72^°^C)
SULTR 4.1	XM_044540797	GCTGTCA CTGGCCTGG TAGATT	CGCTATA GCAATCTGGA TGTCG	40× (10 s 95^°^C, 10 s 63^°^C, 20 s 72^°^C)
				

Primers were synthesized by Sigma-Genosys (Cambridge, United Kingdom). Standardization was carried out based on the expression of the Triticum aestivum RNase L-inhibitor-like protein (RLIa) gene in each sample, using corresponding specific primers (Unigene Ta2776). Data analysis of the relative abundance of the transcripts was done using CFX manager Software Data Analysis (Bio-Rad Laboratories). Data were normalized with respect to the transcript level of the reference gene with the normalized expression method (ΔΔCt). Expression analyses were carried out on three independent root RNA samples and repeated three times for each RNA sample.

### Statistical Analysis

Significant differences (*p* ≤ 0.05) among treatments were calculated by using one-way analysis of variance (ANOVA) and the LSD Fischer *post hoc* test. All statistical tests were performed using the statistical package Statistica 6.0 (StatSoft, Tulsa, OK, United States).

## Results

As explained in materials and methods, two complementary types of experiments were carried out: A first experiment including Fe sufficient- and Fe deficient plants are grown with or without HA in the nutrient solution (referred to as the first experiment in the text). And a second experiment that included Fe sufficient plants and Fe deficient plants, and Fe deficient plants treated with HA or with the Fe concentration contained in HA composition (12.5 μM) (referred to as the second experiment in the text). The results of the two types of experiments are described and discussed in parallel.

### Humic Acid Affects the Growth of Fe-Sufficient and Fe-Deficient Wheat Plants

**FIGURE 1:**
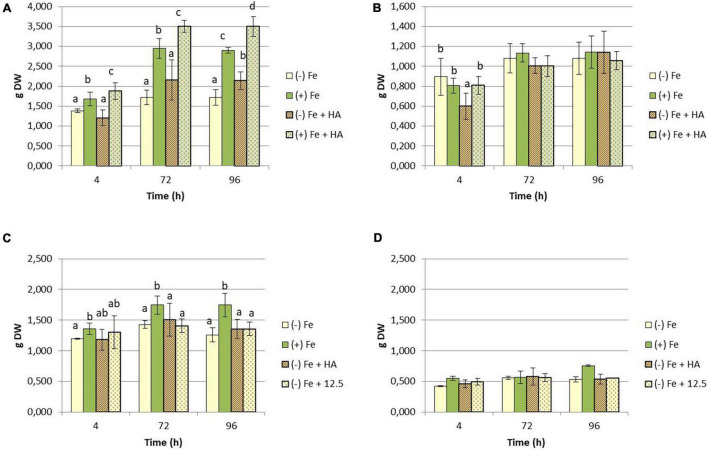
Impact of Fe deficiency and HA treatment on dry matter production expressed in grams (g). **(A)** First experiment. Results for the shoot; **(B)** first experiment. Results for the root *{first experiment treatments: Plants with Fe [(+) Fe]; Plants without Fe [(−) Fe]; plants with Fe plus HA [(+) Fe + HA]; plants without Fe plus HA [(−) Fe + HA)]}*. **(C)** Second experiment. Results for the shoot; **(D)** second experiment. Results for the root *{second experiment treatments: Plants with Fe [(+) Fe]; Plants without Fe [(−) Fe]; plants without Fe plus HA [(−) Fe + HA]; plants plus 12.5 μM Fe [(−) Fe + 12.5]}*. Each data represents the average of three replicates with 23 plants per replicate. Bars represent the standard deviation of the mean (SD). Different letters indicate significant differences between treatments each time (ANOVA followed by an LSD Fischer test, *P* < 0.05).

**FIGURE 2:**
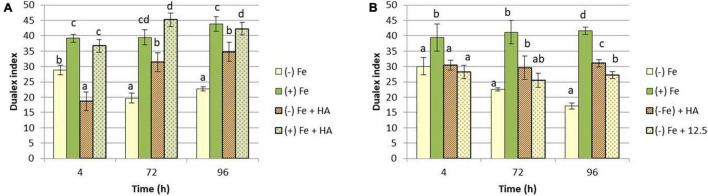
Impact of Fe deficiency and HA on the physiological status of the plants (chlorophyll fluorescence). **(A)** Leaf chlorophyll content expressed as Dualex units for the first experiment *{first experiment treatments: Plants with Fe [(+) Fe]; Plants without Fe [(−) Fe]; plants with Fe plus HA [(+) Fe + HA]; plants without Fe plus HA [(−) Fe + HA]}.* **(B)** Leaf chlorophyll content expressed as Dualex units for the second experiment *{second experiment treatments: Plants with Fe [(+) Fe]; Plants without Fe [(−) Fe]; plants without Fe plus HA [(−) Fe + HA]; plants plus 12.5 μM Fe [(−) Fe + 12.5]}*. Each Dualex data represents the average of 10 measures per leaf and five leaves per replicate in three replicates. Bars represent the standard deviation of the mean (SD). Different letters indicate significant differences between treatments each time (ANOVA followed by an LSD Fischer test, *P* < 0.05).

A number of studies have shown that Fe deficiency significantly impacts crop quality and yields (Barton and Abadia, 2006; Abadía et al., 2011). In our experimental conditions, Fe-starved plants presented a clear reduction in the growth of shoot compared to Fe-fed plants (Figures 1A,C), while no significant differences have been observed in root dry weight (Figures 1B,D). Likewise, intense chlorosis in leaves associated with Fe deficiency was reflected in the decrease in leaf chlorophyll measured in Dualex index (Figures 2A,B).

**FIGURE 3:**
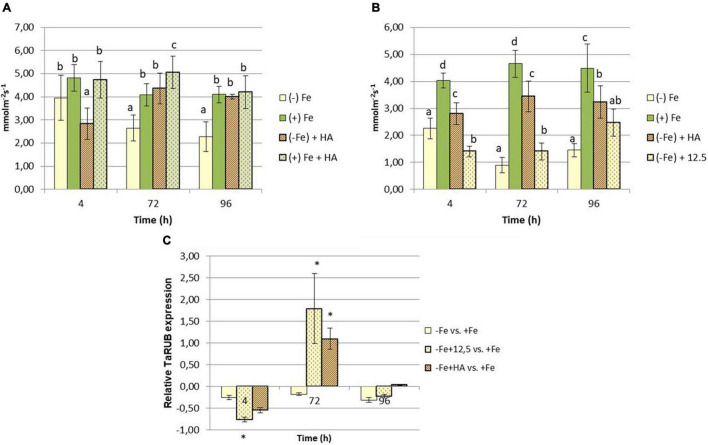
Impact of Fe deficiency and HA on the physiological status of the plants (photosynthetic activity). **(A)** Net photosynthetic rate for the first experiment *{first experiment treatments: Plants with Fe [(+) Fe]; Plants without Fe [(−) Fe]; plants with Fe plus HA [(+) Fe + HA]; plants without Fe plus HA [(−) Fe + HA]}*. **(B)** Net photosynthetic rate for the second experiment *{second experiment treatments: Plants with Fe [(+) Fe]; Plants without Fe [(−) Fe]; plants without Fe plus HA [(−) Fe + HA]; plants plus 12.5 μM Fe [(−) Fe + 12.5]}*. **(C)** Relative root TaRUB gene expression. Each net photosynthetic rate data represents the average of 10 measures per leaf and on five leaves per replicate, in three replicates. Bars represent the standard deviation of the mean (SD). Different letters and * indicate significant differences between treatments each time (ANOVA followed by an LSD Fischer test, *P* < 0.05). Gene expression analyses were carried out on three independent root RNA samples and repeated three times for each RNA sample.

On the other hand, HA application caused a consistent significant increase in shoot growth in Fe sufficient plants and in Fe deficient plants after 96 h (Figures 1A,C). Likewise, HA application caused a significant and consistent increase in net photosynthesis rates in both Fe sufficient and Fe deficient plants (Figures 3A,B). These effects were not caused by the treatment of Fe deficient plants with 12.5 μM of Fe, which presented only a slight increase after 72 and 96 h (Figure 3B). In this line, both HA and 12.5 μM of Fe affected the expression of the gene encoding Rubisco (TaRUB), an enzyme involved in the major step of the photosynthetic process of carbon fixation into ribulose-1,5-bisphosphate. TaRUB was downregulated in all treatments, including Fe deficiency compared with Fe sufficiency, due to the lack of Fe, which induced strong chlorosis in wheat plants (Figure 3C). Nevertheless, 12.5 μM of Fe and HA supply increased its expression significantly after 72 h of treatment, which correlated with an increment of chlorophyll net photosynthetic rate, as stated before (Figure 3C).

In this line, HA also improved the chlorophyll content in leaves of Fe deficient plants, but this effect was similar to that caused by 12.5 μM of Fe (Figures 2A,B).

### Humic Acid Increases the Total Fe- and Soluble Fe-Leaf Concentration in Fe Deficient Plants and the Release of Phytosiderophore From the Root to the Nutrient Solution

**FIGURE 4:**
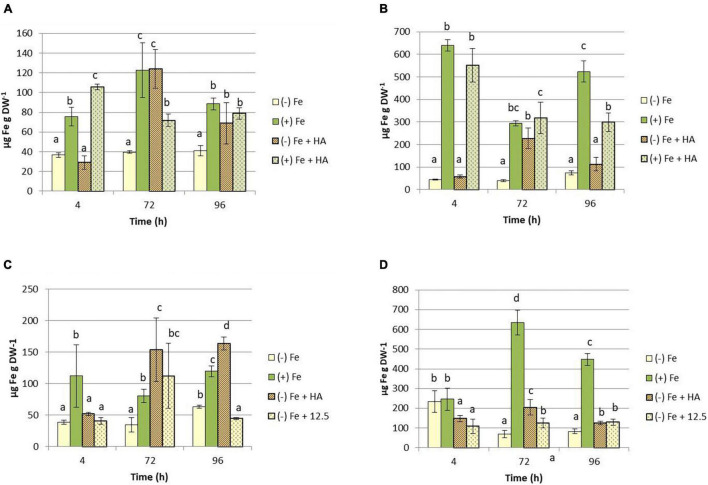
Impact of Fe deficiency and HA on the Fe total concentration in plants. **(A)** Fe total concentration in shoots for the first experiment. **(B)** Fe total concentration in roots for the first experiment. *{first experiment treatments: Plants with Fe [(*+) *Fe]; Plants without Fe [(−) Fe]; plants with Fe plus HA [(*+) *Fe* + *HA]; plants without Fe plus HA [(−) Fe* + *HA)}*. **(C)** Fe total concentration in shoots for the second experiment. **(D)** Fe total concentration in roots for the second experiment *{second experiment treatments: Plants with Fe [(*+) *Fe]; Plants without Fe [(−) Fe]; plants without Fe plus HA [(−) Fe* + *HA]; plants plus 12.5 μM Fe [(−) Fe* + *12.5]}.* Each data represents the average of three replicates with 23 plants per replicate. Bars represent the standard deviation of the mean (SD). Different letters indicate significant differences between treatments each time (ANOVA followed by an LSD Fischer test, *P* < 0.05).

The above-described effects indicate that HA alleviates the harmful physiological effects of Fe deficiency. This fact might be related to changes in the concentration of Fe fractions (total and soluble) in leaves of Fe deficient plants. The results confirmed this hypothesis. HA caused a significant increase in the total concentration of Fe in leaves of plants subjected to Fe deficiency (Figures 4A,C). This effect was higher and more consistent than that caused by 12.5 μM of Fe (Figures 4A,C). This fact was also observed in roots (Figures 4B,D). However, HA did not change the leaf- and root-Fe concentration in Fe sufficient plants (Figure 4A).

**FIGURE 5:**
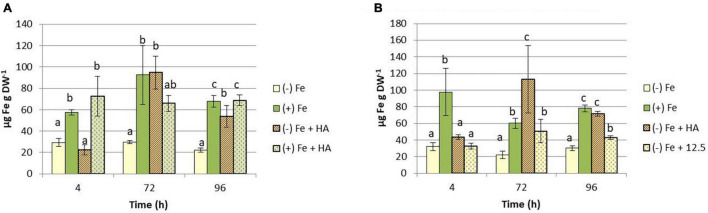
Impact of Fe deficiency and HA on the Fe-soluble concentration in shoots. **(A)** Results for the first experiment *{first experiment treatments: Plants with Fe [(*+) *Fe]; Plants without Fe [(−) Fe]; plants with Fe plus HA [(*+) *Fe* + *HA]; plants without Fe plus HA [(−) Fe* + *HA]}*. **(B)** Results for the second experiment *{second experiment treatments: Plants with Fe [(*+) *Fe]; Plants without Fe [(−) Fe]; plants without Fe plus HA [(−) Fe* + *HA]; plants plus 12.5 μM Fe [(−) Fe* + *12.5]}.* Each data represents the average of three replicates with 23 plants per replicate. Bars represent the standard deviation of the mean (SD). Different letters indicate significant differences between treatments each time (ANOVA followed by an LSD Fischer test, *P* < 0.05).

The effects of HA on the Fe-soluble fraction in leaves were similar to those on Fe-total concentration (Figures 5A,B).

**FIGURE 6:**
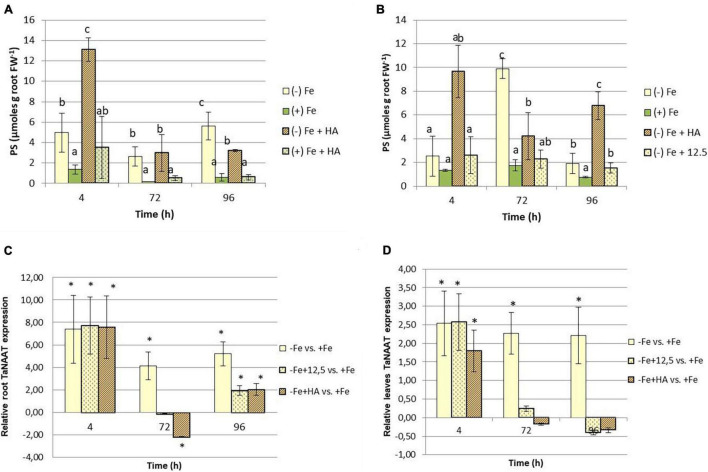
Impact of Fe deficiency and HA on the root release of phytosiderophores. **(A)** Results for the first experiment *{first experiment treatments: Plants with Fe [(+) Fe]; Plants without Fe [(−) Fe]; plants with Fe plus HA [(+) Fe + HA]; plants without Fe plus HA [(−) Fe + HA]}.* **(B)** Results for the second experiment *{second experiment treatments: Plants with Fe [(+) Fe]; Plants without Fe [(−) Fe]; plants without Fe plus HA [(−) Fe + HA]; plants plus 12.5 μM Fe [(−) Fe + 12.5]}*. **(C)** Relative expression of the gene encoding the nicotianamine amino transferase (TaNAAT) in roots. **(D)** Relative expression of the gene encoding the nicotianamine amino transferase (TaNAAT) in shoots. Each data represents the average of three replicates with 23 plants per replicate. Bars represent the standard deviation of the mean (SD). Different letters and * indicate significant differences between treatments each time (ANOVA followed by an LSD Fischer test, *P* < 0.05). Gene expression analyses were carried out on three independent root RNA samples and repeated three times for each RNA sample.

This increase in Fe fractions in leaves caused by HA in Fe deficient plants, principally related to Fe-soluble fraction, suggests that HA might promote the biosynthesis of Fe chelating compounds capable of mobilizing Fe, such as the phytosiderophores. These compounds might react with HA promoting the mobilization of the Fe complexed by HA. In order to investigate this fact, we measured the root release of phytosiderophores to the nutrient solution of the plants included in all treatments. The results clearly show that HA promotes the root phytosiderophore release in Fe deficient plants (Figures 6A,B). This effect is observed after 4 h from the onset of treatments and seems to consist in an increase in the precocity of phytosiderophore release, although the phytosiderophore concentration also increased (Figures 6A,B). This promoting effect is not caused by 12.5 μM of Fe, which presented levels similar or lower than Fe deficient plants (Figure 6B). On the other hand HA did not affect root phytosiderophore release in Fe sufficient plants (Figure 6A).

Recent studies have reported that root phytosiderophore release is associated with the upregulation of the gene encoding the nicotianamine aminotransferase (TaNAAT) in wheat roots (Garnica et al., 2018). The effects of HA promoting root phytosiderophore release may be related to the upregulation of this gene. Our study shows that Fe deficient plants, and their treatment with HA or 12.5 μM of Fe, was linked to a significant upregulation of TaNAAT after 4 h from the onset of the treatments (Figures 6C,D). However, whereas Fe deficient plants maintained the up-regulation at 72 and 96 h, both HA and 12.5 μM of Fe showed some downregulation after 72 h and a slight but significant upregulation after 96 h (Figure 6C). In HA treatment, there was a good concordance between the pattern of variation of TaNAAT regulation and root phytosiderophores release (Figure 6A). This concordance was less clear than in the case of 12.5 μM of Fe treatment (Figure 6B).

As reported above, HA also caused significant increases in the soluble-Fe fraction in leaves (Figures 5A,B). This effect might be related to the presence of molecules with Fe-chelating ability also in leaves. In order to explore this fact, we measured the expression of TaNAAT in leaves. The results showed that Fe deficient plants and their treatment with HA and 12.5 μM of Fe presented a slight but significant upregulation of TaNAAT in leaves after 4 h from the onset of the experiment. This result was maintained for 72 and 96 h in Fe deficient plants but not in HA- and 12.5 μM of Fe- treated Fe deficient plants (Figure 6D).

### Humic Acid Affects the Nitrate Concentration in the Leaves of Fe Sufficient and Deficient Wheat Plants

**FIGURE 7:**
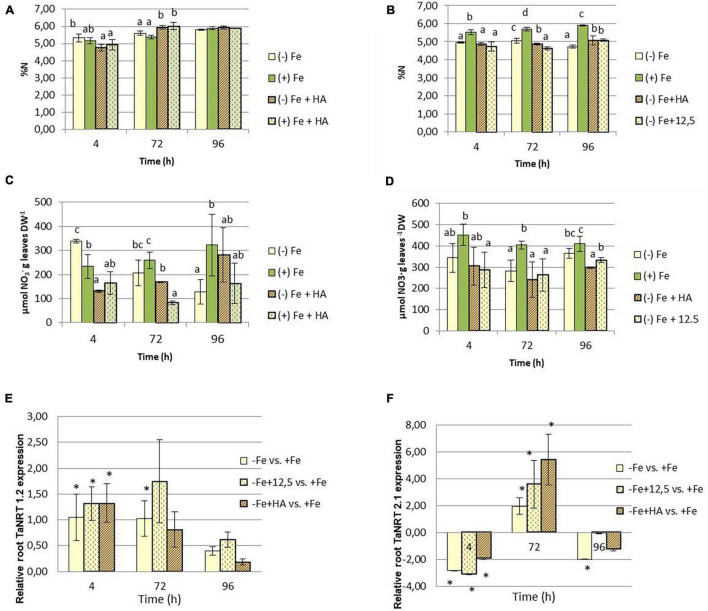
Impact of Fe deficiency and HA on the N fractions within the plant. **(A)** Total N concentration in shoots for the first experiment. **(B)** Total N concentration in roots for the first experiment. **(C)** Nitrate concentration in shoots for the first experiment *{first experiment treatments: Plants with Fe [(*+) *Fe]; Plants without Fe [(−) Fe]; plants with Fe plus HA [(*+) *Fe* + *HA]; plants without Fe plus HA [(−) Fe* + *HA]}*. **(D)** Nitrate concentration in shoots for the second experiment *(second experiment treatments: Plants with Fe [(*+) *Fe]; Plants without Fe [(−) Fe]; plants without Fe plus HA [(−) Fe* + *HA]; plants plus 12.5 μM Fe [(−) Fe* + *12.5]*}. **(E)** Relative expression of the gene encoding NRT1.2 nitrate transporter in roots. **(F)** Relative expression of the gene encoding NRT2.1 nitrate transporter in roots. Each data represents the average of three replicates with 23 plants per replicate. Bars represent the standard deviation of the mean (SD). Different letters or * indicate significant differences between treatments each time (ANOVA followed by an LSD Fischer test, *P* < 0.05). Gene expression analyses were carried out on three independent root RNA samples and repeated three times for each RNA sample.

Several studies have shown the ability of HS to promote nitrate root uptake, and its further conversion in ammonium by the action of nitrate reductase, in several plant species (Nardi et al., 2002; Mora et al., 2010; Jannin et al., 2012; Tavares et al., 2017). Our results show that whereas the effects of HA on total N concentration in leaves are small and transient (only for 72 h) (Figure 7A), the effects on nitrate are much more straightforward (Figures 7C,D). In general, HA treatment reduces nitrate concentration in leaves after 4 and 72 h from the onset of treatments (Figure 7C). This effect was more pronounced than that of 12.5 μM of Fe at 96 h, which presented the same pattern of variation as that of Fe deficient plants (Figure 7D). It was noteworthy that Fe deficiency as well as Fe deficient plants treated with HA and 12.5 μM of Fe showed a significant decrease of nitrate concentration in roots, principally after 72 years 96 h from the onset of treatments.

The study of the expression in roots of the genes codifying the nitrate transporters TaNRT1.2 and TaNRT2.1 show that Fe deficiency is associated with the upregulation of TaNRT1.2 after 4 and 72 h from the onset of treatments (Figure 7E). However, HA- and 12.5 μM of Fe-treatment of Fe deficient plants only upregulated this gene at 4 h (Figure 7E). Fe-deficient plants and their treatment with HA and 12.5 μM of Fe downregulated TaNRT2.1 at 4 h and upregulated this gene at 72 h (Figure 7F).

### Humic Acid Affects the Concentration of Mineral Elements of Fe Sufficient and Fe-Deficient Wheat Plants

**FIGURE 8:**
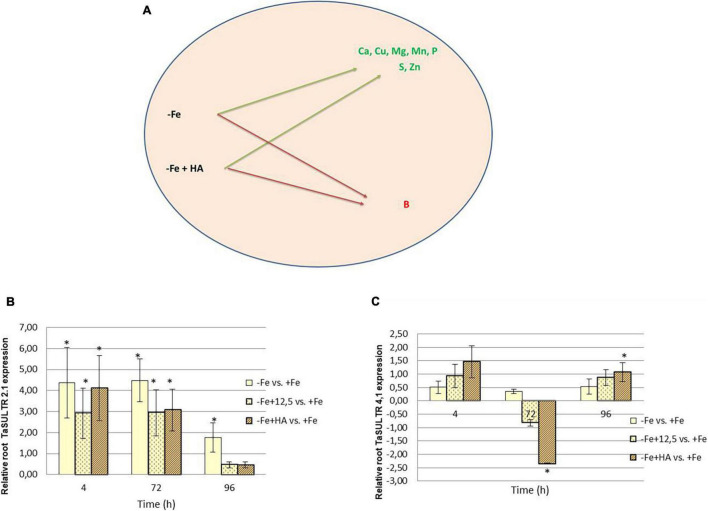
Impact of Fe deficiency and HA on the plant ionome. **(A)** Mineral elements affected by Fe deficiency and HA treatment. **(B)** Relative expression of the gene encoding SULTR2.1 sulfate transporter in roots. **(C)** Relative expression of the gene encoding SULTR4.1 sulfate transporter in roots. * indicates significant differences between treatments each time (ANOVA followed by an LSD Fischer test, *P* < 0.05). Gene expression analyses were carried out on three independent root RNA samples and repeated three times for each RNA sample.

Several studies have reported that Fe deficiency, on the one hand, and HS treatment, on the other hand, can modify the concentration of other nutrients in both leaves and roots (Mora et al., 2010; Olaetxea et al., 2018; D’Oria et al., 2021). Our results regarding the concentration of mineral elements (except Fe and N) of Fe deficient- and Fe sufficient plants grown with or without HA in the nutrient solution indicate that the effect of Fe deficiency is preponderant compared with the effect of HA treatment (Tables 1, 2 and Figure 8A). As there were no noticeable differences between the results obtained in the first and second experiments, the results presented here correspond to the first experiment.

**TABLE 1:**
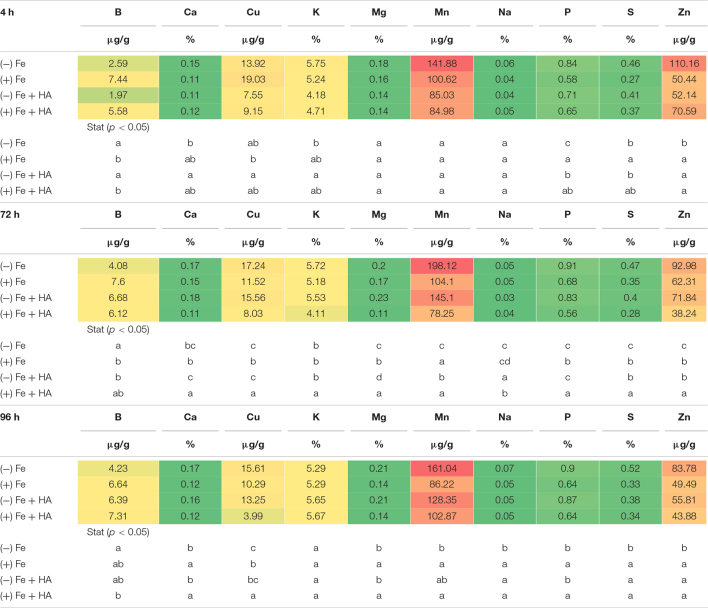
Impact of Fe deficiency and HA on the partial leaf ionome.

*Each data represents the average of three replicates with 23 plants per replicate. Different letters indicate significant differences between treatments for each time (ANOVA followed by a LSD Fischer test, P < 0.05). In heatmap, the color is related to the concentration value with red colors corresponding to the higher values and green colors to the lowest ones.*

**TABLE 2:**
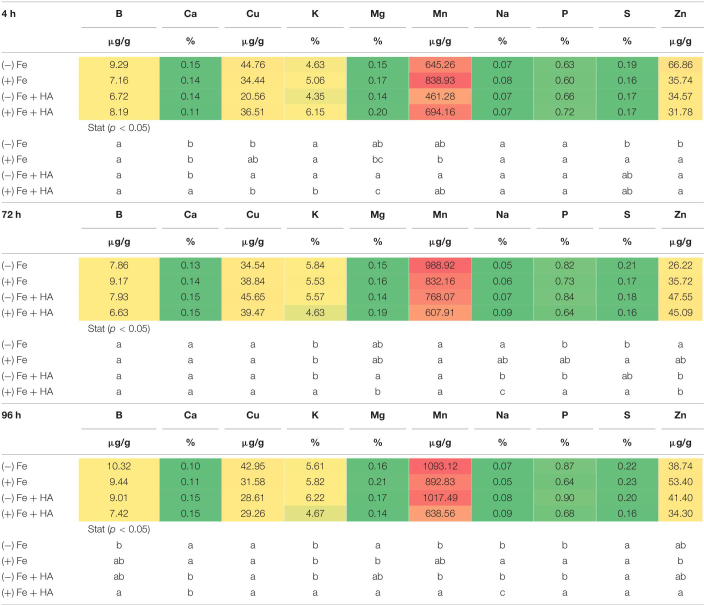
Impact of Fe deficiency and HA on the partial root ionome.

*Each data represents the average of three replicates with 23 plants per replicate. Different letters indicate significant differences between treatments for each time (ANOVA followed by a LSD Fischer test, P < 0.05). In heatmap, the color is related to the concentration value with red colors corresponding to the higher values and green colors to the lowest ones.*

Fe deficiency and Fe deficiency plus HA caused an increase in the shoot concentration of Ca (96 h), Cu (72 and 96 h), Mg (96 h), Mn (72, 96 h), S (4, 72, less clear at 96 h), Zn (4, 72 h, less clear at 96 h) and P (4, 72, 96 h) (Table 1 and Figure 8A). On the other hand, these treatments caused a decrease in the shoot concentration of B (Table 1).

The results in the root were less clear than those in the shoot, but Fe deficiency and Fe deficiency plus HA caused an increase in the concentration of Mn (96 h); and P (96 h), while Fe deficiency alone caused a decrease in the concentration of Mg (96 h) (Table 2).

Previous studies in other plant species reported that HS induced the upregulation of some sulfate transporters in the root, with this effect being correlated to the sulfate concentration in the shoot (Jannin et al., 2012). In this experiment, we have analyzed two sulfate transporters, TaSULTR 2.1, a low-affinity transporter, and TaSULTR4.1, a transporter involved in vacuolar efflux, in the roots of Fe deficient plants and their treatment with HA and 12.5 μM of Fe, compared with Fe sufficient plants (Figure 8B). In general, Fe deficiency was associated with the upregulation of TaSULTR 2.1 at 4 and 72 h. However, this effect was significantly reduced after 96 h with HA and 12.5 μM of Fe supply (Figure 8B). On the other hand, TaSULTR4.1 was downregulated by HA at 72 h and slightly upregulated at 4 and 96 h (Figure 8C).

### Humic Acid Modifies the Hormonal Balance of Fe Sufficient and Fe Deficient Wheat Plants

Several studies have reported that the action of several phytohormones regulates the root response of dicotyledonous plants to Fe deficiency, principally ethylene, IAA, NO, and ABA (Bacaicoa et al., 2009, 2011; García et al., 2010, 2011; Lei et al., 2014; Zhang et al., 2020); but also salicylic acid (SA) (Shen et al., 2016), jasmonic acid (JA) (Kobayashi et al., 2016; Cui et al., 2018) and CKs (Séguéla et al., 2008). However, the studies in the graminaceous plants are scarce (Garnica et al., 2018; Divte et al., 2019). Garnica et al. (2018) reported in wheat that IAA regulates the release of phytosiderophores from the root to the rhizosphere in plants grown under Fe deficiency, but not by ethylene, as well as by the Fe status in the shoot. Divte et al. (2019) reported in wheat that ethylene affects phytosiderophore biosynthesis within the root.

**FIGURE 9:**
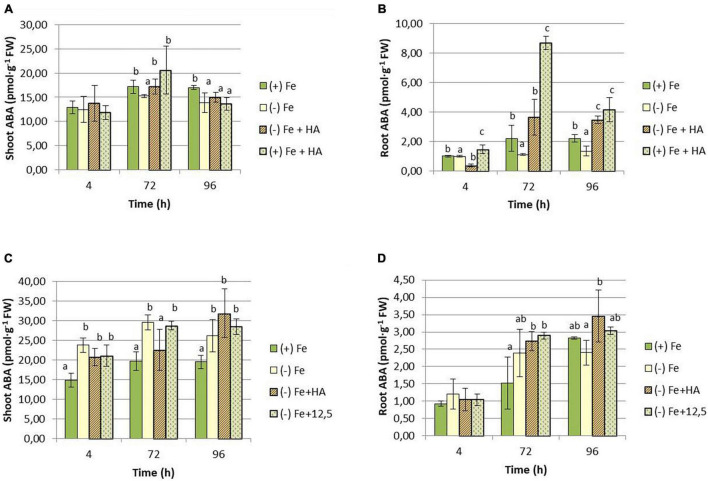
Impact of Fe deficiency and HA on ABA concentration in the plant. **(A)** Results in shoots for the first experiment. **(B)** Results in roots for the first experiment *{first experiment treatments: Plants with Fe [(*+) *Fe]; Plants without Fe [(−) Fe]; plants with Fe plus HA [(*+) *Fe* + *HA]; plants without Fe plus HA [(−) Fe* + *HA]}*. **(C)** Results in shoots for the second experiment. **(D)** Results in roots for the second experiment *{second experiment treatments: Plants with Fe [(*+) *Fe]; Plants without Fe [(−) Fe]; plants without Fe plus HA [(−) Fe* + *HA]; plants plus 12.5 μM Fe [(−) Fe* + *12.5]}.* Each data represents the average of three replicates with 23 plants per replicate. Bars represent the standard deviation of the mean (SD). Different letters indicate significant differences between treatments each time (ANOVA followed by an LSD Fischer test, *P* < 0.05).

Our study shows that Fe deficiency affected the concentration of ABA in both root and shoot (Figure 9). In the case of the shoot, we only observe an increase in Fe deficient plants in the second experiment that included 12.5 μM of Fe treatment (Figures 9A,C). This inconsistency between the two types of experiments (first experiment and second experiment) could be related to the presence of plants with different development stages between the two experiments that may affect the variation pattern of hormone concentrations over time. There were no differences between Fe deficient plants and their treatment with HA and 12.5 μM of Fe (Figure 9C). However, the results in roots are more consistent (Figures 9B,D). HA supply to Fe sufficient plants caused an increase in ABA concentration compared with Fe sufficient- and deficient-control plants (Figure 9B). However, there were no differences between Fe deficient plants and their treatment with HA and 12.5 μM of Fe (Figure 9C).

**FIGURE 10:**
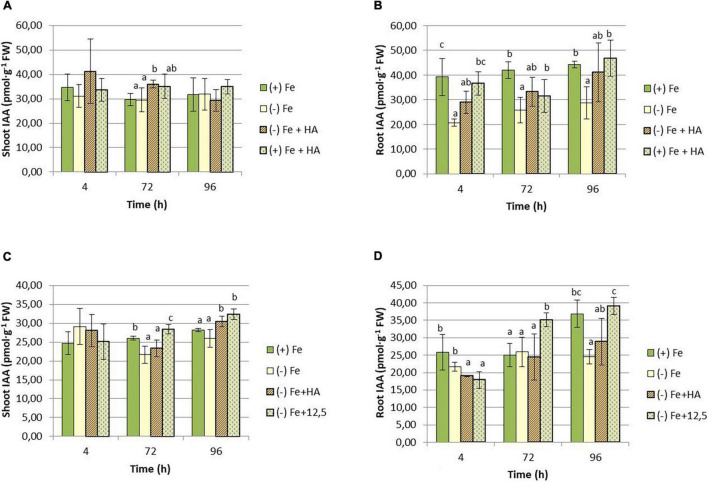
Impact of Fe deficiency and HA on IAA concentration in the plant. **(A)** Results in shoots for the first experiment. **(B)** Results in roots for the first experiment *{first experiment treatments: Plants with Fe [(*+) *Fe]; Plants without Fe [(−) Fe]; plants with Fe plus HA [(*+) *Fe* + *HA]; plants without Fe plus HA [(−) Fe* + *HA]}*. **(C)** Results in shoots for the second experiment. **(D)** Results in roots for the second experiment *{second experiment treatments: Plants with Fe [(*+) *Fe]; Plants without Fe [(−) Fe]; plants without Fe plus HA [(−) Fe* + *HA]; plants plus 12.5 μM Fe [(−) Fe* + *12.5]}.* Each data represents the average of three replicates with 23 plants per replicate. Bars represent the standard deviation of the mean (SD). Different letters indicate significant differences between treatments each time (ANOVA followed by an LSD Fischer test, *P* < 0.05).

Regarding IAA, both HA and 12.5 μM of Fe caused a slight increase in IAA shoot concentration compared with Fe deficient plants at 72 h (Figure 10A) and 96 h (Figure 10C). However, in roots, Fe deficiency caused a consistent decrease in the concentration of IAA (Figures 10B,D).

**FIGURE 11:**
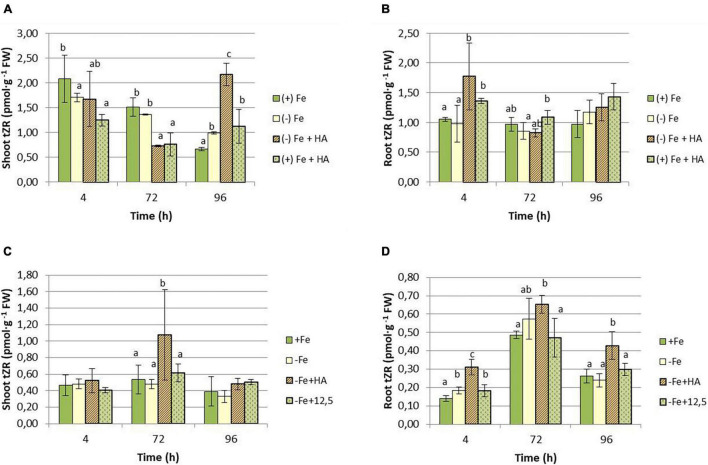
Impact of Fe deficiency and HA on tZR concentration in the plant. **(A)** Results in shoots for the first experiment. **(B)** Results in roots for the first experiment *{first experiment treatments: Plants with Fe [(*+) *Fe]; Plants without Fe [(−) Fe]; plants with Fe plus HA [(*+) *Fe* + *HA]; plants without Fe plus HA [(−) Fe* + *HA]}*. **(C)** Results in shoots for the second experiment. **(D)** Results in roots for the second experiment *{second experiment treatments: Plants with Fe [(*+) *Fe]; Plants without Fe [(−) Fe]; plants without Fe plus HA [(−) Fe* + *HA]; plants plus 12.5 μM Fe [(−) Fe* + *12.5]}.* Each data represents the average of three replicates with 23 plants per replicate. Bars represent the standard deviation of the mean (SD). Different letters indicate significant differences between treatments each time (ANOVA followed by an LSD Fischer test, *P* < 0.05).

As for CKs, the results show that HA supply increased the shoot concentration of tZR in Fe deficient plants compared with their control of Fe deficient plants (Figures 11A,C). This effect was observed at 96 h in the first experiment (Figure 11A) and 72 h in the second experiment (Figure 11C). The effects of HA supply were not observed in 12.5 μM of Fe supply (Figure 11C). There was also an increase in tZR in HA-treated Fe sufficient plants compared with Fe sufficient control plants at 96 h, preceded by a reduction at 4 and 72 h (Figure 11A). In roots, HA supply to Fe deficient plants produced an increase in tZR compared with Fe deficient control plants at 4 h for the first experiment (Figure 11B) and at 4 and 96 h for the second experiment (Figure 11D). As observed in the shoot, the effects caused by HA supply were not observed for 12.5 μM of Fe (Figure 11D).

**FIGURE 12:**
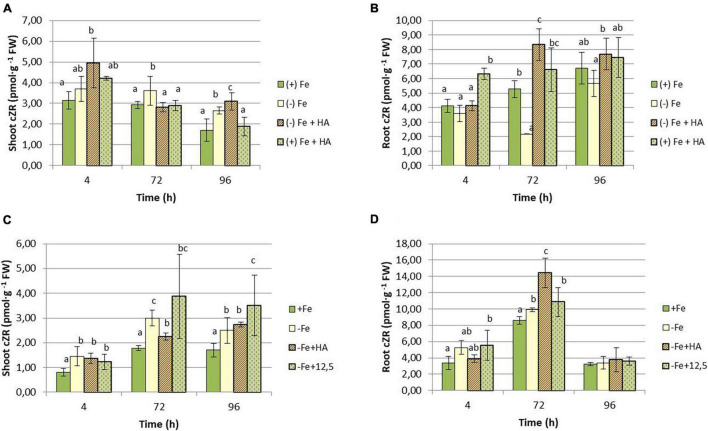
Impact of Fe deficiency and HA on cZR concentration in the plant. **(A)** Results in shoots for the first experiment. **(B)** Results in roots for the first experiment *{first experiment treatments: Plants with Fe [(*+) *Fe]; Plants without Fe [(−) Fe]; plants with Fe plus HA [(*+) *Fe* + *HA]; plants without Fe plus HA [(−) Fe* + *HA]}*. **(C)** Results in shoots for the second experiment. **(D)** Results in roots for the second experiment *{second experiment treatments: Plants with Fe [(*+) *Fe]; Plants without Fe [(−) Fe]; plants without Fe plus HA [(−) Fe* + *HA]; plants plus 12.5 μM Fe [(−) Fe* + *12.5]}.* Each data represents the average of three replicates with 23 plants per replicate. Bars represent the standard deviation of the mean (SD). Different letters indicate significant differences between treatments each time (ANOVA followed by an LSD Fischer test, *P* < 0.05).

As for cZR, Fe deficiency caused a decrease in the shoot concentration of this hormone (Figures 12A,C). Although HA supply to Fe deficient plants seems to potentiate this effect, the results were not clear due to data variability (Figures 12A,C). HA supply to Fe sufficient plants showed a slight increase in cZR in leaves compared with Fe sufficient control plants at 4 h only (Figure 12A). In roots, however, HA supply to Fe deficient plants caused a clear increase of cZR compared with Fe deficient control plants at 72 and 96 h in the first experiment (Figure 12B) and 72 h in the second experiment (Figure 12D). This effect was not observed for 12.5 μM of Fe (Figure 12D).

As for iP, Fe deficiency increased the concentration of this hormone in the shoot (Supplementary Figures S3.1A,C). HA or 12.5 μM of Fe treatments did not change this effect of Fe deficiency (Supplementary Figure S3.1C). The results in the root did not show a clear pattern of variation (Supplementary Figures S3.1B,D).

The pattern of variation of iPR was similar to that of iP (Supplementary Figures S3.2A–D).

## Discussion

### Humic Acid Alleviates the Deleterious Physiological Effects of Fe Deficiency by Increasing Fe Bioavailability Within the Plant, and Promoting Phytosiderophore Root Release and Production

The root application of HA, a humic acid extracted and purified from a black peat substrate, promoted the growth of wheat plants cultivated in hydroponics and without any Fe limitation (Figure 1A). This effect is in line with the results reported for many studies on the ability of HS to promote plant growth (Rose et al., 2014; Olaetxea et al., 2018; Nardi et al., 2021). Likewise, HA could improve the growth of Fe deficient wheat plants after 96 h from the onset of treatments (Figures 1A,C). In this line, the plant physiological status of Fe deficient plants treated with HA clearly improved as it was reflected in significant increases in chlorophyll content and net photosynthetic rates (Figures 2A, 3A). In addition, control Fe deficient plants supplied with the concentration of Fe contained in HA composition (12.5 μM Fe) did not experience these effects at the same level as that of Fe deficient plants treated with HA (Figures 2B, 3B). This fact is compatible with different but complementary mechanisms. On the one hand it might reflect that the Fe complexed by HA is used by wheat plants more efficiently than that chelated by EDTA. On the other hand it may reflect a direct action of the organic moiety of HA.

The results obtained concerning the Fe distribution within the plant indicate that the two mechanisms could be acting together. Thus, HA caused significant increases in the leaf concentration of total Fe and, more interestingly, soluble (more active) Fe (Figures 4A,B, 5A). However, the 12.5 μM Fe control treatment did not cause these effects at the same level as that of HA, consistently with the effects on chlorophyll and photosynthetic rates discussed above (Figures 4C,D, 5B).

One possible explanation is that HA-treated plants produce and release from the root to the nutrient solution Fe chelating compounds, such as the phytosiderophores. These compounds might interact with the Fe complexed by HA promoting its root uptake and further translocation. In this sense, the results obtained in the microarray study of Fe sufficient wheat plants treated with HA are meaningful (Supplementary Annex 2). Although the function of most of the genes affected by HA treatment were unknown and the information obtained is quite limited, several genes related to Fe uptake, phytosiderophore biosynthesis and Fe transport were upregulated in the roots of wheat plants (nicotianamine and nicotianamine amino transferase activity).

Our results confirmed this hypothesis since HA clearly potentiated the short-term release of phytosiderophores from the root to the nutrient solution in Fe deficient plants compared with non-HA treated Fe deficient plants (Figure 6A). Consistently, 12.5 μM Fe control treatment did not cause this effect, thus indicating that it is ascribed to HA in itself. In line with these results, whereas Fe deficiency caused a sustained strong upregulation of the gene codifying the nicotianamine amino transferase (TaNAAT) in roots and shoots (Figures 6C,D), HA-treated deficient plants only presented this strong upregulation at 4 h (Figures 6C,D). The 12.5 μM Fe control treatment presented, in this case, a pattern of variation similar to that of HA (Figures 6C,D). In both cases, the results reflected the alleviating action of the two treatments on Fe deficiency consequences. However, the beneficial action of HA was much more intense than that of 12.5 μM Fe control treatment.

In this framework, the effect of HA promoting root phytosiderophores release would be previous to the increase in Fe fractions in the shoot (Fe-independent) and linked to some feature (or features) of its organic structure and molecular conformation in solution. Previous studies in wheat showed that the release of phytosiderophores from the root to the nutrient solution is regulated by the hormonal balance within the plant (Garnica et al., 2018). It is thus possible that the action of HA on this process is also regulated by HA-induced changes of the hormonal balance.

### The Beneficial Action of Humic Acid on the Physiological Status of Fe Deficient Plants Is Associated With Significant Increases of *Trans*-Zeatin Riboside in Roots and Shoots and *cis*-Zeatin Riboside in Roots

As mentioned above, HS can activate the root response to Fe deficiency in dicot, both under Fe deficiency and Fe sufficiency (Aguirre et al., 2009). Although the mechanism responsible for this effect remains unclear, it might be related to the ability of HS to increase the root concentration of IAA, NO, and ethylene (Zandonadi et al., 2010, 2013; Mora et al., 2012, 2014; Olaetxea et al., 2015, 2019). Several studies have shown that these three phytohormones are directly involved in activating Fe deficiency root responses in dicots (Bacaicoa et al., 2009, 2011; García et al., 2010, 2011; García-Mina et al., 2013). Recent studies have reported that the root release of phytosiderophores to the rhizosphere in wheat is regulated by IAA although any change in its concentration in roots was observed (Garnica et al., 2018). In line with the results reported by Garnica et al. (2018), we did not observe an increase in IAA root concentration in Fe deficient wheat plants but a decrease (Figure 11). These results suggest that the regulation mediated by IAA might be related to changes in the distribution of IAA in the root or some IAA-aminoacid conjugates’ action (Garnica et al., 2018). We observed a slight increase of root-IAA in HA-treated Fe deficient plants. However, this action was similar to that of 12.5 μM Fe, indicating that this is not related to both the physiological improvement and the increase in root phytosiderophore release caused by HA in Fe deficient plants. However, if the regulation of IAA on phytosiderophore root release is mediated by other factors different from its total concentration in roots, an action of IAA in the effect of HA cannot be ruled out.

Some studies have shown that the root concentration of ABA in roots modulates root responses to Fe deficiency in dicots (Lei et al., 2014; Zhang et al., 2020). In our study, we observed that HA caused a slight and transient increase in Fe sufficient plants in shoots (Figure 9A) and a strong increase in roots (Figure 9B). On the other hand, Fe deficiency caused an increase in shoots (Figure 9C). This increase was observed in Fe deficient plants treated with HA and 12.5 μM Fe (Figure 9D). This fact indicates that this effect on ABA is linked to Fe deficiency, and it probably does not influence the HA effects on Fe homeostasis and root phytosiderophore release.

Several studies have reported the very relevant role of the root to shoot translocation of some types of CKs (mainly tZ and tZR but also iP and iPR), linked to nitrate root uptake and further translocation, in the promoting effect of HS on shoot growth and root to shoot nutrient translocation (Mora et al., 2010; Olaetxea et al., 2019). Several studies have reported the crucial role of tZR and tZ in the long-distance signaling system controlling plant growth and nutrient root acquisition (Osugi et al., 2017; Sakakibara, 2021). In our study, whereas the effects on iP and iPR seem to be specific of Fe deficiency without being influenced by HA, the effects on tZR and cZR are very specific of HA. Thus, HA caused a clear increase in the shoot concentration of tZR in Fe- deficient and sufficient plants (Figures 11A,C). This effect of HA was observed in Fe deficient plants in both roots and shoots (Figures 11A–D), while 12.5 μM Fe did not cause any effect. This effect on tZR mediated by HA in Fe deficient plants was accompanied by an increase in cZR in the roots of these plants. Some studies have described the signaling role of the ratio between tZ and cZ in regulating the root response to the deficiency of some nutrients, such as P and N (Osugi et al., 2017; Silva-Navas et al., 2019; Sakakibara, 2021). In this framework, it would be possible that the two CKs are also involved in the beneficial action of HA on the development of Fe deficiency root responses in graminaceous. Indeed, as discussed above, the effect of HA on tZR and cZR in plants suffering Fe deficiency is specific of HA in itself, and it could be related to the enhanced root to shoot translocation of Fe fractions caused by HA in Fe deficient plants. However, mechanistic studies involving inhibitors of CKs biosynthesis and/or function, or specific mutants, are needed to elucidate this question.

In dicots, CK application to the roots caused an inhibition of the activation of root responses to Fe deficiency to adapt plant growth to Fe availability (Séguéla et al., 2008). Our results suggest that these phytohormones do not play the same role in graminaceous if the above discussed effects of HA on tZR and cZR are integrated in the beneficial action of HA on Fe availability. If that is not the case, our results might be the consequence of HA-mediated Fe chlorosis alleviation (the increase in the shoot concentration of Fe fractions), driving further plant growth (Séguéla et al., 2008).

Finally, a potential role of NO is also plausible since, on the one hand, NO regulates Fe remobilization within the plant in either dicots or monocots (Graziano et al., 2002), and on the other hand, HS enhances NO production in several species (Zandonadi et al., 2010; Mora et al., 2012).

### Both Fe Deficiency and Humic Acid Affected the Concentration of Mineral Nutrients in Root and Shoot of Fe Deficient Plants

As discussed above, several studies have reported that HS application promotes nitrate root uptake and further transformation in ammonium due to an increase in the leaf nitrate reductase activity (Mora et al., 2010; Jannin et al., 2012). In our experiment, we do not observe very relevant effects of Fe deficiency or HA application on the concentration of total N in the shoot. However, the application of HA in Fe sufficient plants reduced shoot nitrate (Figures 7C,D). This effect has been observed in several plant species and is due to the enhancement of nitrate reductase activity (Mora et al., 2010; Jannin et al., 2012). On the other hand, Fe deficiency tended to decrease the concentration of nitrate in the shoots, with this effect being potentiated by the presence of HA (Figures 7C,D). In line with previous studies that showed the ability of HS to induce the upregulation of the genes encoding the main nitrate transporters in the root of several plant species (Nardi et al., 2002; Jannin et al., 2012; Tavares et al., 2017; Zanin et al., 2018), the HA-mediated effects on nitrate leaf concentration were associated with the upregulation of the expression of TaNRT 1.2 and TaNRT 2.1 nitrate transporters in roots of Fe deficient plants (Figures 7E,F). In the case of TaNRT 1.2, this effect was similar to that caused by Fe deficiency alone. However, TaNRT2.1 expression was upregulated by HA in Fe deficient plant roots up to higher levels than those associated with Fe deficiency alone and 12.5 μM Fe (Figures 7E,F). Considering that this nitrate transporter is associated with nitrate transport in plant tissues, this specific effect of HA is likely associated with nitrate distribution within the plant. In fact the upregulation of TaNRT2.1 after 72 h caused by Fe deficiency, and HA and 12.5 μM Fe in Fe deficient plants, is associated with a decrease of nitrate concentration in the root after 72 and 96 h (Figure 7D). This effect was linked to a reduction of total N in the root but not in the shoot (Figures 7A,B), thus suggesting an increase in nitrate root to shoot translocation. However, further experiments using labeled ^15^N are needed in order to confirm this fact. The reduction in nitrate concentration in the root above described was not linked to concomitant increases of nitrate concentration in the shoot (Figure 7D). Considering that there were not differences between treatments regarding total N (Figure 7A), this fact suggests that the activity of nitrate reductase in the shoot increased in Fe deficient plants treated with HA. Likewise, taking into account the close co-regulation of the root to shoot translocation of both nitrate and t-Z cytokinin family (Sakakibara, 2021), this effect might be also linked to the increase in t-ZR mediated by HA in Fe-deficient plants.

Baxter et al. (2008) showed that the leaf ionome can be used as an effective tool for diagnosing plant physiological status. Using Arabidopsis plants, they observed that Fe deficiency status can be detected by using several elements as positive markers, such as Mn, Co, Zn, Mo, and Cd. Recently, D’Oria et al. (2021) observed in wheat that Fe deficiency led to increases in the relative net uptake of several nutrients, such as Co, Si, Mo, Zn, Cu, Ni, Ca, S, and Mg. In our case, Fe deficiency was associated with systematic increases in the leaf concentration of some micronutrients (Cu, Zn, and Mn), some secondary nutrients (Ca, Mg, and S), and one macronutrient (P) (Table 1). This effect was also associated with a decrease in B leaf concentration (Table 1), also reported by D’Oria et al. (2021). The main difference between D’Oria’s results (D’Oria et al., 2021) and our results is related to P that experienced a significant increase in shoot and root (96 h) with Fe deficiency in our study. On the other hand, several studies have reported that Fe deficiency upregulated the expression of some of the genes involved in P root uptake (Zanin et al., 2017; Lucena et al., 2019). This fact might explain the increase of leaf-P concentration associated with Fe deficiency observed in our experiments.

The application of HA to Fe deficient plants did not change the leaf-mineral concentration pattern associated with Fe deficiency (Table 1). Previous studies have described that HS may induce the upregulation of the expression of specific transporters related to micronutrient root uptake and sulfate (Jannin et al., 2012; Billard et al., 2014). In our study, we observed that Fe deficiency induced the upregulation of the expression of SULTR 2.1 in roots (Figure 8B). This effect was also caused by HA and 12.5 μM Fe in Fe deficient plant roots, although this upregulation disappeared with time. However, in SULTR 4.1 the upregulation caused by HA was more consistent than those of Fe deficiency alone and 12.5 μM Fe (Figure 8C). This transporter regulates sulfate efflux from the vacuole to the cytoplasm (Saito, 2004). The effect of HA on the regulation of its expression suggests an action of HA on sulfate remobilization within the plant.

## Conclusion

In summary, the results presented here show that HA can improve the physiological status of Fe deficient wheat plants by alleviating some of the deleterious consequences of Fe deficiency on plant development. This action of HA is associated with increases in the Fe-active pool in leaves as well as the plant ability to, presumably produce, and release phytosiderophores from the root to the rhizosphere. The results indicate that the two HA-mediated effects might be coordinated, with the increase in Fe fractions in the shoot resulting from the interaction of the Fe complexed in HA with the phytosiderophores released by the root. Fe root to shoot translocation may be favored by the action oftZR since the concentration of this phytohormone was enhanced by HA in the leaves of Fe deficient plants.

**FIGURE 13:**
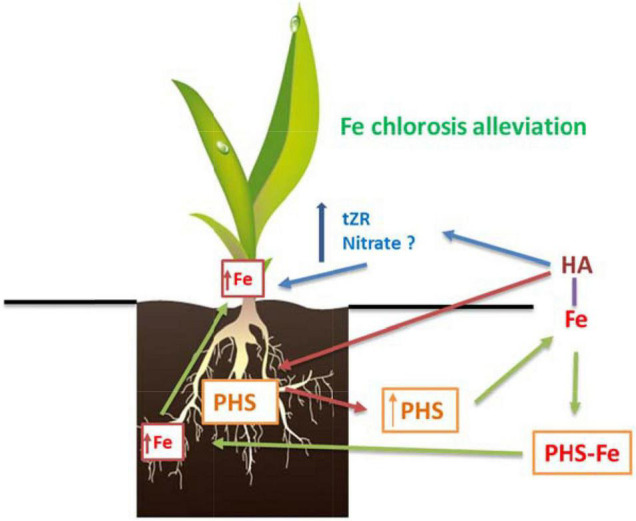
Proposed mechanism for the beneficial action of HA on graminaceous plants suffering Fe deficiency. First, HA promotes the release of phytosiderophores (PHS) to the nutrient solution. Second, PHS interact with the Fe complexed in HA promoting its absorption by the roots. Third, there is an increase in the concentrations of total-Fe and active-Fe in the leaves that is related to Fe chlorosis alleviation. Fourth, the Fe root to shoot translocation could be favored by the HA-mediated increase in tZR in leaves.

Therefore, the alleviating effect of HA in Fe deficient plants seem to combine mechanisms both dependent and independent of the Fe complexed by HA (Figure 13).

## Data Availability Statement

All datasets generated in this study are included in the article/Supplementary Material.

## Author Contributions

MG, RB, and JG-M planned and designed the research. MG, RB, and SS carried out the plant experiments and analytical measurements. MG carried out the gene expression study. AZ carried out the hormonal analysis. MG, RB, SS, and JG-M critically analyzed the results. MG and JG-M prepared the manuscript. All authors read and approved the final manuscript.

## Conflict of Interest

The authors declare that the research was conducted in the absence of any commercial or financial relationships that could be construed as a potential conflict of interest.

## Publisher’s Note

All claims expressed in this article are solely those of the authors and do not necessarily represent those of their affiliated organizations, or those of the publisher, the editors and the reviewers. Any product that may be evaluated in this article, or claim that may be made by its manufacturer, is not guaranteed or endorsed by the publisher.
