# Supplementary material for: Humic Acid Alleviates Fe Chlorosis in Graminaceous Plants Through Coordinated Fe-Dependent and Fe-Independent Mechanisms
Source: Front Plant Sci. 2022 Feb 2;13:803013. doi: 10.3389/fpls.2022.803013 (PMC8849133; doi:10.3389/fpls.2022.803013)
Supplement: Supplementary file 1 [file Data_Sheet_1.docx]

**Supplementary Material**

A peat-derived humic acid alleviates Fe chlorosis deleterious effects in graminaceous plants by improving Fe usage and phytosiderophore root release, in association with an enhancement in the concentration of trans zeatin riboside and cis zeatin riboside in plant tissues.

**ANNEX 1. Physicochemical characterization of the humic acid (HA)**


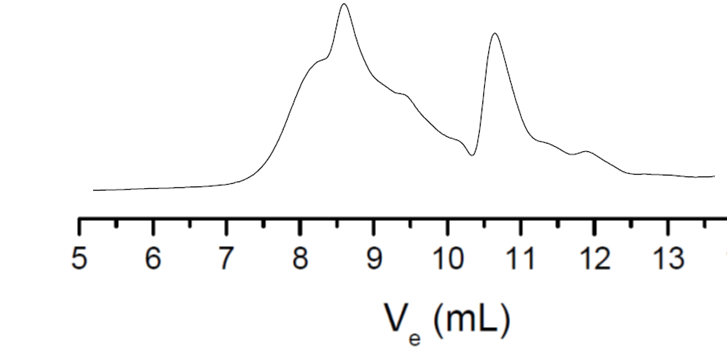


**Figure S1.** Size-distribution HPSEC chromatogram for HA

**Table S1**. Molecular weight distribution of HA, obtained from Figure S1, expressed either as the MW corresponding to the maximum of the main peaks, or as the MW corresponding to the interval of the whole peak.

| **Sample** | **Main peaks (Da)** | **Interval MW (Da)** | **Área (%)** |
| --- | --- | --- | --- |
| HA | 2,68·10^4^ | 1,21·10^4^ – 6,88·10^4^ | 21 |
|  | 1,39·10^4^ | 9,67·10^3^ – 2,02·10^4^ | 11 |
|  | 8,26·10^3^ | 1,41·10^3^ – 5,07·10^4^ | 65 |
|  | 1,89·10^3^ | 9,65·10^2^ – 3,43·10^3^ | 3 |


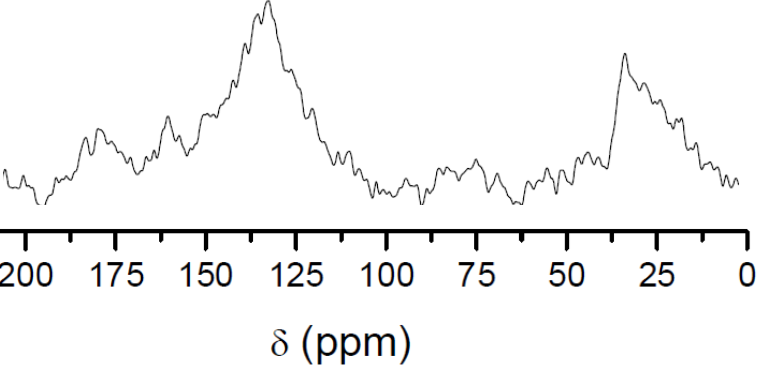


**Figure S2.**  ^13^C NMR spectra of HA

**Table S2.** Relative abundances of different carbon types (in %) determined by ^13^C NMR for HA.

|  | Alkyl C | O-alkyl C | Aromatic C | Phenolic C | Carboxylic C | Carbonylic C |
| --- | --- | --- | --- | --- | --- | --- |
| *δ (ppm)* | *0-45* | *45-110* | *110-160* | *140-160* | *160-185* | *185-215* |
| E7 | 27.7 | 16.7 | 42.0 | 7.8 | 8.2 | 5.4 |

**ANNEX 2. Microarray analysis**

A previous microarray study was carried out in wheat plants. The plants were cultivated as described in Material and Methods. This study was designed to investigate the effect of HA on the gene expression of wheat plants cultivated under conditions of Fe sufficiency.

The experiment consisted of two treatments: A control with plants growing under Fe sufficiency and without HA treatment, and the HA treatment with plants growing under Fe sufficiency and treated with HA (100 mg C L-1). The plants were harvested after 24h, 72h and 30 days from the onset of treatments. Samples of roots and shoots were frozen in liquid N and send to Imaxio laboratory (Lyon, France) for RNA extraction and microarray analysis.

**Methodology**

Each test sample is hybridized on Wheat Gene Expression Microarray 4 X 44K (Agilent Technologies) using two-color microarray-based gene expression protocol. In this procedure, the controls and the test samples are respectively labeled with cyanine 3 and cyanine 5. For each vegetal part and each time, the same control is used for the different hybridizations. For this, the three RNA control replicates are pooled, and then twice labeled with cyanine 3.

The Agilent RNA Spike-In Kit was developed to provide positive controls for monitoring the microarray workflow from sample amplification and labeling to microarray processing. The two colors RNA Spikes-In Kit contains two spike-in mixtures. In each mixture, 10 in vitro synthesized, polyadenylated transcripts derived from the Adenovirus E1A are premixed at various ratios. These controls are labeled and amplified together with the RNA samples.

Sample labeling: Low Input Quick Amp Labeling Kit (Agilent Technologies) generates fluorescent cRNA with 200 ng of total RNA. A primer containing poly dT and a T7 polymerase promoter is annealed to the poly A+ RNA. Reverse transcriptase is added to the reaction to synthesize the first and second strands of cDNA. Next, cRNA is synthesized from the double-stranded cDNA using T7 RNA polymerase, which simultaneously incorporates cyanine-labeled CTP. Before proceeding to the sample hybridization, labeled cRNA and dye concentration are quantified and validated.

After the labeling step, cRNA sample size ranges from 50 to 3000 nucleotides. Thus, fragmentation is required to take away secondary structures. Fragmentation step is achieved with specific buffer and allows obtaining cRNA length between 50 to 200 nucleotides and then optimal hybridization with Agilent 60-mer oligonucleotide microarrays. This step is an incubation at 60°C for 30 min.

Hybridizations are performed at 65°C for 17 h. Scanning is performed with the Agilent scanner using default parameters for 4 x 44 K formats. For an XDR extraction, two image files (.tiff) are generated, one noted H (high, PMT 100) and one noted L (low, PMT 10). Data are extracted with Feature Extraction 10.1 software (Agilent Technologies). This software reads and processes microarray image files to prepare them for analysis. Feature Extraction automatically assigns a grid template and a protocol, based on the barcode of the slide. It determines feature intensities, rejects outliers and calculates statistical confidences.

**Results**

Microarray (Array Express: E-MTAB-11280) revealed that only the results corresponding to the roots of 72 h harvest plants time presented a significant modification of the gene expression as a consequence of HA treatment (fold change greater than 2.0 and T-test p< 0.05 **Annex2**).

545 genes were upregulated with 34 being functionally characterized (6.2 %): ABA: 4; Jasmonic acid: 2; Aminoglicanes: 1; Nitrogen: 8; Carbon/photosynthesis: 2; phosphorus: 7; Transferase activity: 1; cell metabolism: 9.

1202 genes were downregulated with 269 being functionally characterized (22.4 %): ABA and Gibberellins: 19; Jasmonic acid: 3; Aminoglicanes: 2; Nitrogen: 21; Aminoacid cell metabolism: 11; Carbon/photosynthesis 144; phosphorus: 56; Transferase activity: 6; cell metabolism: 7. (See attached file for complete raw data.)

Although the function of most of the genes affected by HA treatment were unknown and the information obtained is quite limited, several genes related to Fe uptake, phytosiderophore biosynthesis and Fe transport were upregulated in the roots of wheat plants (nicotianamine and nicotianamine amino transferase activity).

**Table S2.1**

***Functionally characterized downregulated genes (p<0.05) for the roots of wheat plants treated with HA after 72 h from the onset of the treatment.***

| ***Description*** | **Fold Change** |
| --- | --- |
|  |  |
| abscisic acid mediated signaling pathway;hormone-mediated signaling pathway;cellular response to hormone stimulus;response to hormone stimulus;response to abscisic acid stimulus;cellular response to abscisic acid stimulus | 2.7629144 |
| abscisic acid mediated signaling pathway;hormone-mediated signaling pathway;cellular response to hormone stimulus;response to hormone stimulus;response to abscisic acid stimulus;cellular response to abscisic acid stimulus | 2.7558398 |
| abscisic acid mediated signaling pathway;hormone-mediated signaling pathway;cellular response to hormone stimulus;response to hormone stimulus;response to abscisic acid stimulus;cellular response to abscisic acid stimulus | 2.6454303 |
| abscisic acid mediated signaling pathway;hormone-mediated signaling pathway;cellular response to hormone stimulus;response to hormone stimulus;response to abscisic acid stimulus;cellular response to abscisic acid stimulus | 2.4670367 |
| aminoglycan catabolic process;aminoglycan metabolic process;nitrogen compound metabolic process | 3.9811597 |
| aminoglycan catabolic process;aminoglycan metabolic process;nitrogen compound metabolic process | 2.5193446 |
| Azote | 7.5252542 |
| Azote | 3.9618535 |
| Azote | 2.7821093 |
| Azote | 2.46142 |
| Azote | 2.344649 |
| Azote | 2.2916489 |
| Azote | 2.1842215 |
| Azote | 2.171866 |
| Azote | 2.022168 |
| Azote | 2.0122058 |
| Azote;cellular amino acid metabolic process;glutamine family amino acid metabolic process;glutamate decarboxylase activity;cellular amino acid and derivative metabolic process;glutamate metabolic process;cellular nitrogen compound metabolic process;nitrogen compound metabolic process | 2.1270292 |
| Azote;chloroplast thylakoid;photosynthesis, light reaction;chloroplast thylakoid membrane;chloroplast part;photosynthesis;chloroplast | 15.651076 |
| Azote;detection of ethylene stimulus;cellular amino acid metabolic process;response to ethylene stimulus;sulfur amino acid metabolic process;cellular amino acid derivative metabolic process;1-aminocyclopropane-1-carboxylate oxidase activity;detection of hormone stimulus;ethylene metabolic process;response to hormone stimulus;cellular amino acid and derivative metabolic process;cellular amino acid derivative biosynthetic process;ethylene biosynthetic process;cellular nitrogen compound metabolic process;aspartate family amino acid metabolic process;nitrogen compound metabolic process | 2.8731194 |
| cellular amino acid derivative metabolic process;cellular amino acid and derivative metabolic process | 2.3986425 |
| cellular amino acid derivative metabolic process;cellular amino acid and derivative metabolic process | 2.3167763 |
| cellular amino acid derivative metabolic process;cellular amino acid and derivative metabolic process;cellular amino acid derivative biosynthetic process | 5.2034354 |
| cellular amino acid derivative metabolic process;cellular amino acid and derivative metabolic process;cellular amino acid derivative biosynthetic process | 3.8675563 |
| cellular amino acid derivative metabolic process;cellular amino acid and derivative metabolic process;cellular amino acid derivative biosynthetic process | 3.460421 |
| cellular amino acid derivative metabolic process;cellular amino acid and derivative metabolic process;cellular amino acid derivative biosynthetic process | 2.9884171 |
| cellular amino acid derivative metabolic process;cellular amino acid and derivative metabolic process;cellular amino acid derivative biosynthetic process | 2.9245903 |
| cellular amino acid derivative metabolic process;cellular amino acid and derivative metabolic process;cellular amino acid derivative biosynthetic process | 2.4355834 |
| cellular amino acid metabolic process;aromatic amino acid family biosynthetic process;hormone biosynthetic process;regulation of hormone levels;response to ethylene stimulus;cellular amino acid derivative metabolic process;hormone metabolic process;tryptophan metabolic process;response to hormone stimulus;cellular amino acid and derivative metabolic process;cellular nitrogen compound biosynthetic process;cellular amino acid derivative biosynthetic process;aromatic amino acid family metabolic process;tryptophan biosynthetic process;chloroplast;cellular nitrogen compound metabolic process;cellular amino acid biosynthetic process;nitrogen compound metabolic process | 2.0559456 |
| cellular amino acid metabolic process;cellular amino acid catabolic process;cellular amino acid and derivative metabolic process;cellular nitrogen compound metabolic process;aspartate family amino acid metabolic process;aspartate family amino acid catabolic process;nitrogen compound metabolic process | 2.357346 |
| cellular nitrogen compound metabolic process;nucleoside phosphate metabolic process;nitrogen compound metabolic process | 2.7448342 |
| chloroplast stroma;chloroplast part;chloroplast | 18.457659 |
| chloroplast thylakoid;chloroplast thylakoid membrane;chloroplast part;cellular nitrogen compound biosynthetic process;chloroplast;chlorophyll metabolic process;chlorophyll biosynthetic process;cellular nitrogen compound metabolic process;nitrogen compound metabolic process | 3.5503201 |
| chloroplast thylakoid;chloroplast thylakoid membrane;chloroplast part;chloroplast | 4.2240005 |
| chloroplast thylakoid;chloroplast thylakoid membrane;chloroplast part;chloroplast | 3.2688897 |
| chloroplast thylakoid;chloroplast thylakoid membrane;chloroplast stroma;chloroplast part;photosynthesis;chloroplast | 190.06093 |
| chloroplast thylakoid;chloroplast thylakoid membrane;chloroplast stroma;chloroplast part;photosynthesis;chloroplast | 56.834602 |
| chloroplast thylakoid;chloroplast thylakoid membrane;chloroplast stroma;chloroplast part;photosynthesis;chloroplast | 40.394012 |
| chloroplast thylakoid;jasmonic acid biosynthetic process;chloroplast thylakoid membrane;jasmonic acid metabolic process;chloroplast part;chloroplast | 5.622983 |
| chloroplast thylakoid;jasmonic acid biosynthetic process;chloroplast thylakoid membrane;jasmonic acid metabolic process;chloroplast part;chloroplast | 5.3555503 |
| chloroplast thylakoid;phosphorylation;phosphate metabolic process;chloroplast thylakoid membrane;phosphorus metabolic process;chloroplast part;oxidative phosphorylation;chloroplast | 2.674661 |
| chloroplast thylakoid;photosynthesis, light reaction;chlorophyll binding;chloroplast thylakoid membrane;chloroplast stroma;chloroplast part;photosynthesis, light harvesting;photosynthesis;chloroplast;photosynthesis, light harvesting in photosystem II | 89.69844 |
| chloroplast thylakoid;photosynthesis, light reaction;chlorophyll binding;chloroplast thylakoid membrane;chloroplast stroma;chloroplast part;photosynthesis, light harvesting;photosynthesis;chloroplast;photosynthesis, light harvesting in photosystem II | 25.396835 |
| chloroplast thylakoid;photosynthesis, light reaction;chloroplast part;photosynthesis;chloroplast | 22.918213 |
| chloroplast thylakoid;photosynthesis, light reaction;chloroplast thylakoid membrane;chloroplast part;photosynthesis;chloroplast | 58.788746 |
| chloroplast thylakoid;photosynthesis, light reaction;chloroplast thylakoid membrane;photosynthesis, light harvesting in photosystem I;chloroplast stroma;chloroplast part;photosynthesis, light harvesting;photosynthesis;chloroplast | 76.738846 |
| chloroplast thylakoid;photosynthesis, light reaction;chloroplast thylakoid membrane;photosynthesis, light harvesting in photosystem I;chloroplast stroma;chloroplast part;photosynthesis, light harvesting;photosynthesis;chloroplast | 33.691833 |
| chloroplast thylakoid;photosynthesis, light reaction;chloroplast thylakoid membrane;photosynthesis, light harvesting in photosystem I;chloroplast stroma;chloroplast part;photosynthesis, light harvesting;photosynthesis;chloroplast | 21.24481 |
| chloroplast thylakoid;photosynthesis, light reaction;chloroplast thylakoid membrane;photosynthesis, light harvesting in photosystem I;chloroplast stroma;chloroplast part;photosynthesis, light harvesting;photosynthesis;chloroplast | 14.887678 |
| glutamate biosynthetic process;glutamate synthase activity;glutamate synthase activity, NADH or NADPH as acceptor;cellular amino acid metabolic process;glutamine family amino acid metabolic process;glutamine family amino acid biosynthetic process;cellular amino acid and derivative metabolic process;cellular nitrogen compound biosynthetic process;glutamate metabolic process;cellular nitrogen compound metabolic process;cellular amino acid biosynthetic process;nitrogen compound metabolic process | 2.3425455 |
| Hormone | 3.496431 |
| Hormone | 3.2637315 |
| hydrolase activity, acting on carbon-nitrogen (but not peptide) bonds;hydrolase activity, acting on carbon-nitrogen (but not peptide) bonds, in linear amides;protein amino acid deacetylation;cellular nitrogen compound metabolic process;nitrogen compound metabolic process | 18.670519 |
| jasmonic acid biosynthetic process;jasmonic acid metabolic process | 4.7722926 |
| jasmonic acid biosynthetic process;jasmonic acid metabolic process;response to jasmonic acid stimulus;response to hormone stimulus;response to abscisic acid stimulus;root system development;root development | 10.937045 |
| jasmonic acid biosynthetic process;jasmonic acid metabolic process;response to jasmonic acid stimulus;response to hormone stimulus;response to abscisic acid stimulus;root system development;root development | 2.2989693 |
| ligase activity, forming carbon-nitrogen bonds;acid-amino acid ligase activity | 3.4801593 |
| negative regulation of nitrogen compound metabolic process;hydrolase activity, acting on carbon-nitrogen (but not peptide) bonds;pyrophosphatase activity;hydrolase activity, acting on carbon-nitrogen (but not peptide) bonds, in linear amides;nucleoside-triphosphatase activity;protein amino acid deacetylation;regulation of nitrogen compound metabolic process;hydrolase activity, acting on acid anhydrides, in phosphorus-containing anhydrides;cellular nitrogen compound metabolic process;nitrogen compound metabolic process | 28.868814 |
| negative regulation of nitrogen compound metabolic process;phosphorylation;ionotropic glutamate receptor binding;phosphate metabolic process;peptidyl-amino acid modification;peptidyl-serine phosphorylation;phosphorus metabolic process;transferase activity, transferring phosphorus-containing groups;protein amino acid phosphorylation;regulation of nitrogen compound metabolic process;glutamate receptor binding;cellular nitrogen compound metabolic process;phosphotransferase activity, alcohol group as acceptor;nitrogen compound metabolic process | 2.1786177 |
| organophosphate metabolic process;phospholipid biosynthetic process;phosphatidylserine decarboxylase activity;phospholipid metabolic process | 2.0957165 |
| peptidase activity, acting on L-amino acid peptides | 2.7835858 |
| peptidase activity, acting on L-amino acid peptides | 2.6857312 |
| peptidyl-amino acid modification;cellular amino acid derivative metabolic process;cellular amino acid and derivative metabolic process;nitrogen compound metabolic process | 2.1610801 |
| phospholipid binding;phosphatidylethanolamine binding | 2.3095143 |
| phosphoprotein phosphatase activity;protein serine/threonine phosphatase activity;phosphatase activity;phosphoric ester hydrolase activity | 2.071379 |
| phosphoprotein phosphatase activity;response to hormone stimulus;cellular nitrogen compound biosynthetic process;protein tyrosine phosphatase activity;pentose-phosphate shunt, oxidative branch;chloroplast;pentose-phosphate shunt;cellular nitrogen compound metabolic process;phosphatase activity;nucleoside phosphate metabolic process;phosphoric ester hydrolase activity;nitrogen compound metabolic process | 3.0967736 |
| Phosphore | 40.930786 |
| Phosphore | 22.694885 |
| Phosphore | 10.080887 |
| Phosphore | 10.021216 |
| Phosphore | 9.967096 |
| Phosphore | 9.866236 |
| Phosphore | 9.498971 |
| Phosphore | 9.450388 |
| Phosphore | 9.351449 |
| Phosphore | 9.332124 |
| Phosphore | 9.300332 |
| Phosphore | 9.110128 |
| Phosphore | 8.671094 |
| Phosphore | 8.658213 |
| Phosphore | 8.605096 |
| Phosphore | 8.531446 |
| Phosphore | 8.496306 |
| Phosphore | 8.016272 |
| Phosphore | 5.508215 |
| Phosphore | 5.0620723 |
| Phosphore | 4.960269 |
| Phosphore | 4.5633693 |
| Phosphore | 4.349672 |
| Phosphore | 4.0596747 |
| Phosphore | 3.8578248 |
| Phosphore | 3.797604 |
| Phosphore | 3.6641471 |
| Phosphore | 3.1154718 |
| Phosphore | 2.810186 |
| Phosphore | 2.728161 |
| Phosphore | 2.528922 |
| Phosphore | 2.1664453 |
| Phosphore | 2.1560013 |
| Phosphore | 2.105885 |
| Phosphore | 2.0805295 |
| Phosphore;chloroplast stroma;chloroplast part;chloroplast;pentose-phosphate shunt;cellular nitrogen compound metabolic process;nucleoside phosphate metabolic process;nitrogen compound metabolic process | 14.176947 |
| Phosphore;chloroplast stroma;chloroplast part;chloroplast;pentose-phosphate shunt;cellular nitrogen compound metabolic process;nucleoside phosphate metabolic process;nitrogen compound metabolic process | 7.295179 |
| Phosphore;chloroplast stroma;chloroplast part;chloroplast;pentose-phosphate shunt;cellular nitrogen compound metabolic process;nucleoside phosphate metabolic process;nitrogen compound metabolic process | 7.260983 |
| Phosphore;chloroplast stroma;chloroplast part;chloroplast;pentose-phosphate shunt;cellular nitrogen compound metabolic process;nucleoside phosphate metabolic process;nitrogen compound metabolic process | 7.1983514 |
| Phosphore;chloroplast stroma;chloroplast part;chloroplast;pentose-phosphate shunt;cellular nitrogen compound metabolic process;nucleoside phosphate metabolic process;nitrogen compound metabolic process | 7.000731 |
| Phosphore;chloroplast stroma;chloroplast part;fructose-bisphosphate aldolase activity;chloroplast;pentose-phosphate shunt;cellular nitrogen compound metabolic process;nucleoside phosphate metabolic process;nitrogen compound metabolic process | 3.236368 |
| Phosphore;fructose-2,6-bisphosphate 2-phosphatase activity;carbohydrate phosphatase activity;fructose 1,6-bisphosphate 1-phosphatase activity;phosphatase activity;phosphoric ester hydrolase activity | 3.2378006 |
| Phosphore;phosphate transport;inorganic phosphate transmembrane transporter activity;phosphate transmembrane transporter activity | 2.1878653 |
| Phosphore;photosynthesis, dark reaction;regulation of nitrogen compound metabolic process;triose-phosphate isomerase activity;photosynthesis;chloroplast;cellular nitrogen compound metabolic process;reductive pentose-phosphate cycle;nitrogen compound metabolic process | 2.5604725 |
| Phosphore;pyrophosphatase activity;inorganic diphosphatase activity;hydrolase activity, acting on acid anhydrides, in phosphorus-containing anhydrides | 2.201353 |
| Phosphore;transferase activity, transferring phosphorus-containing groups;phosphotransferase activity, alcohol group as acceptor | 2.0030158 |
| phosphorylation;phosphate metabolic process;phosphorus metabolic process;transferase activity, transferring phosphorus-containing groups;protein amino acid phosphorylation;phosphotransferase activity, alcohol group as acceptor | 8.086901 |
| phosphorylation;phosphate metabolic process;phosphorus metabolic process;transferase activity, transferring phosphorus-containing groups;protein amino acid phosphorylation;phosphotransferase activity, alcohol group as acceptor | 4.7892547 |
| phosphorylation;phosphate metabolic process;phosphorus metabolic process;transferase activity, transferring phosphorus-containing groups;protein amino acid phosphorylation;phosphotransferase activity, alcohol group as acceptor | 3.2797163 |
| phosphorylation;phosphate metabolic process;phosphorus metabolic process;transferase activity, transferring phosphorus-containing groups;protein amino acid phosphorylation;phosphotransferase activity, alcohol group as acceptor | 3.2344675 |
| phosphorylation;phosphate metabolic process;phosphorus metabolic process;transferase activity, transferring phosphorus-containing groups;protein amino acid phosphorylation;phosphotransferase activity, alcohol group as acceptor | 2.95072 |
| phosphorylation;phosphate metabolic process;phosphorus metabolic process;transferase activity, transferring phosphorus-containing groups;protein amino acid phosphorylation;phosphotransferase activity, alcohol group as acceptor | 2.3898253 |
| Photosynthesis | 83.27105 |
| Photosynthesis | 67.84088 |
| Photosynthesis | 54.342533 |
| Photosynthesis | 52.7997 |
| Photosynthesis | 51.442753 |
| Photosynthesis | 50.738888 |
| Photosynthesis | 47.63129 |
| Photosynthesis | 45.57817 |
| Photosynthesis | 40.0729 |
| Photosynthesis | 39.71908 |
| Photosynthesis | 36.211815 |
| Photosynthesis | 35.03297 |
| Photosynthesis | 34.22344 |
| Photosynthesis | 33.14283 |
| Photosynthesis | 31.170164 |
| Photosynthesis | 29.263943 |
| Photosynthesis | 27.717644 |
| Photosynthesis | 23.277243 |
| Photosynthesis | 22.976856 |
| Photosynthesis | 18.13619 |
| Photosynthesis | 14.902409 |
| Photosynthesis | 13.497484 |
| photosynthesis | 11.693664 |
| Photosynthesis | 11.546589 |
| Photosynthesis | 11.246854 |
| Photosynthesis | 10.7662945 |
| Photosynthesis | 10.608835 |
| Photosynthesis | 10.418197 |
| Photosynthesis | 9.648807 |
| Photosynthesis | 9.581558 |
| Photosynthesis | 9.473833 |
| Photosynthesis | 9.351684 |
| Photosynthesis | 9.199219 |
| Photosynthesis | 8.8231945 |
| Photosynthesis | 8.713738 |
| Photosynthesis | 8.53601 |
| Photosynthesis | 8.110441 |
| Photosynthesis | 7.859032 |
| Photosynthesis | 7.8215003 |
| Photosynthesis | 7.771627 |
| Photosynthesis | 5.580765 |
| Photosynthesis | 5.1205893 |
| Photosynthesis | 5.005204 |
| Photosynthesis | 4.8320656 |
| Photosynthesis | 4.6164474 |
| Photosynthesis | 4.5275435 |
| Photosynthesis | 4.176469 |
| Photosynthesis | 3.9846897 |
| Photosynthesis | 3.879188 |
| Photosynthesis | 3.4358413 |
| Photosynthesis | 3.308476 |
| Photosynthesis | 3.2874434 |
| Photosynthesis | 3.194062 |
| photosynthesis | 2.7141974 |
| Photosynthesis | 2.6314185 |
| Photosynthesis | 2.360795 |
| Photosynthesis | 2.0409007 |
| Photosynthesis;chloroplast thylakoid;chloroplast thylakoid membrane;chloroplast part;chloroplast | 60.932663 |
| Photosynthesis;chloroplast thylakoid;chloroplast thylakoid membrane;chloroplast part;chloroplast | 2.6925027 |
| Photosynthesis;chloroplast thylakoid;chloroplast thylakoid membrane;chloroplast stroma;chloroplast part;chloroplast | 32.790638 |
| Photosynthesis;chloroplast thylakoid;chloroplast thylakoid membrane;chloroplast stroma;chloroplast part;chloroplast stromal thylakoid;chloroplast | 32.549496 |
| Photosynthesis;chloroplast thylakoid;chloroplast thylakoid membrane;chloroplast stroma;chloroplast part;chloroplast stromal thylakoid;chloroplast | 19.207857 |
| Photosynthesis;chloroplast thylakoid;chloroplast thylakoid membrane;chloroplast stroma;chloroplast part;photosynthesis;chloroplast | 35.501884 |
| Photosynthesis;chloroplast thylakoid;chloroplast thylakoid membrane;chloroplast stroma;chloroplast part;photosynthesis;chloroplast | 15.68059 |
| Photosynthesis;chloroplast thylakoid;chloroplast thylakoid membrane;chloroplast stroma;chloroplast part;photosynthesis;chloroplast | 8.968081 |
| Photosynthesis;chloroplast thylakoid;chloroplast thylakoid membrane;phosphoribulokinase activity;chloroplast part;transferase activity, transferring phosphorus-containing groups;chloroplast;phosphotransferase activity, alcohol group as acceptor | 65.751434 |
| Photosynthesis;chloroplast thylakoid;chloroplast thylakoid membrane;phosphoribulokinase activity;chloroplast part;transferase activity, transferring phosphorus-containing groups;chloroplast;phosphotransferase activity, alcohol group as acceptor | 25.340616 |
| Photosynthesis;chloroplast thylakoid;photosynthesis, light reaction;chlorophyll binding;chloroplast thylakoid membrane;chloroplast stroma;chloroplast part;photosynthesis, light harvesting;photosynthesis;chloroplast;photosynthesis, light harvesting in photosystem II | 84.15689 |
| Photosynthesis;chloroplast thylakoid;photosynthesis, light reaction;chlorophyll binding;chloroplast thylakoid membrane;chloroplast stroma;chloroplast part;photosynthesis, light harvesting;photosynthesis;chloroplast;photosynthesis, light harvesting in photosystem II | 59.923115 |
| Photosynthesis;chloroplast thylakoid;photosynthesis, light reaction;chlorophyll binding;chloroplast thylakoid membrane;chloroplast stroma;chloroplast part;photosynthesis, light harvesting;photosynthesis;chloroplast;photosynthesis, light harvesting in photosystem II | 37.48543 |
| Photosynthesis;chloroplast thylakoid;photosynthesis, light reaction;chlorophyll binding;chloroplast thylakoid membrane;chloroplast stroma;chloroplast part;photosynthesis, light harvesting;photosynthesis;chloroplast;photosynthesis, light harvesting in photosystem II | 20.510845 |
| Photosynthesis;chloroplast thylakoid;photosynthesis, light reaction;chlorophyll binding;chloroplast thylakoid membrane;chloroplast stroma;chloroplast part;photosynthesis, light harvesting;photosynthesis;chloroplast;photosynthesis, light harvesting in photosystem II | 9.420064 |
| Photosynthesis;chloroplast thylakoid;photosynthesis, light reaction;chloroplast thylakoid membrane;chloroplast part;photosynthesis;chloroplast | 46.554764 |
| Photosynthesis;chloroplast thylakoid;photosynthesis, light reaction;chloroplast thylakoid membrane;chloroplast part;photosynthesis;chloroplast | 23.599777 |
| Photosynthesis;chloroplast thylakoid;photosynthesis, light reaction;chloroplast thylakoid membrane;chloroplast part;photosynthesis;chloroplast | 17.691982 |
| Photosynthesis;chloroplast thylakoid;photosynthesis, light reaction;chloroplast thylakoid membrane;chloroplast part;photosynthesis;chloroplast | 14.888106 |
| Photosynthesis;chloroplast thylakoid;photosynthesis, light reaction;chloroplast thylakoid membrane;chloroplast part;photosynthesis;chloroplast | 12.975643 |
| Photosynthesis;chloroplast thylakoid;photosynthesis, light reaction;chloroplast thylakoid membrane;chloroplast part;photosynthesis;chloroplast | 12.002645 |
| Photosynthesis;chloroplast thylakoid;photosynthesis, light reaction;chloroplast thylakoid membrane;chloroplast part;photosynthesis;chloroplast;chloroplast photosystem I;chloroplast thylakoid lumen | 30.86354 |
| Photosynthesis;chloroplast thylakoid;photosynthesis, light reaction;chloroplast thylakoid membrane;chloroplast part;photosynthesis;chloroplast;chloroplast photosystem I;chloroplast thylakoid lumen | 4.941727 |
| Photosynthesis;chloroplast thylakoid;photosynthesis, light reaction;chloroplast thylakoid membrane;chloroplast part;photosynthesis;chloroplast;chloroplast photosystem II;chloroplast thylakoid lumen | 6.5535755 |
| Photosynthesis;chloroplast thylakoid;photosynthesis, light reaction;chloroplast thylakoid membrane;chloroplast part;photosynthesis;chloroplast;chloroplast photosystem II;chloroplast thylakoid lumen | 5.9226475 |
| Photosynthesis;photosynthesis | 11.045753 |
| Photosynthesis;photosynthesis | 9.258475 |
| Photosynthesis;photosynthesis | 8.238858 |
| Photosynthesis;photosynthesis | 7.9066696 |
| Photosynthesis;photosynthesis | 7.2312317 |
| Photosynthesis;photosynthesis | 6.594977 |
| Photosynthesis;photosynthesis | 4.9558034 |
| Photosynthesis;photosynthesis | 2.3292272 |
| Photosynthesis;photosynthesis, light reaction;chloroplast stroma;chloroplast part;photosynthesis;chloroplast | 7.341892 |
| Photosynthesis;photosynthesis, light reaction;chloroplast stroma;chloroplast part;photosynthesis;chloroplast | 3.5497594 |
| Photosynthesis;photosynthesis, light reaction;chloroplast stroma;chloroplast part;photosynthesis;chloroplast | 2.8726707 |
| Photosynthesis;photosynthesis, light reaction;photosynthesis, light harvesting in photosystem I;photosynthesis, light harvesting;photosynthesis | 8.874119 |
| Photosynthesis;purine ribonucleoside triphosphate biosynthetic process;phosphorylation;nucleoside triphosphate metabolic process;phosphate metabolic process;purine nucleoside triphosphate biosynthetic process;purine ribonucleoside triphosphate metabolic process;pyrophosphatase activity;phosphorus metabolic process;nucleoside-triphosphatase activity;hydrogen-exporting ATPase activity, phosphorylative mechanism;ribonucleoside triphosphate biosynthetic process;nucleoside triphosphate biosynthetic process;cellular nitrogen compound biosynthetic process;oxidative phosphorylation;purine nucleoside triphosphate metabolic process;hydrolase activity, acting on acid anhydrides, in phosphorus-containing anhydrides;ribonucleoside triphosphate metabolic process;cellular nitrogen compound metabolic process;ATPase activity, coupled to transmembrane movement of ions, phosphorylative mechanism;nucleoside phosphate metabolic process;nitrogen compound metabolic process | 2.5002801 |
| Photosynthesis;pyrophosphatase activity;nucleoside-triphosphatase activity;hydrolase activity, acting on acid anhydrides, in phosphorus-containing anhydrides | 2.1956654 |
| protein phosphorylated amino acid binding;chloroplast stroma;chloroplast part;phosphoprotein binding;chloroplast;amino acid binding | 4.110526 |
| pyrophosphatase activity;nucleoside-triphosphatase activity;peptidase activity, acting on L-amino acid peptides;hydrolase activity, acting on acid anhydrides, in phosphorus-containing anhydrides | 2.2472675 |
| pyrophosphatase activity;nucleoside-triphosphatase regulator activity;nucleoside-triphosphatase activity;hydrolase activity, acting on acid anhydrides, in phosphorus-containing anhydrides | 3.0234637 |
| regulation of nitrogen compound metabolic process;cellular nitrogen compound metabolic process;nitrogen compound metabolic process | 9.404789 |
| regulation of nitrogen compound metabolic process;cellular nitrogen compound metabolic process;nitrogen compound metabolic process | 8.25427 |
| regulation of nitrogen compound metabolic process;cellular nitrogen compound metabolic process;nitrogen compound metabolic process | 5.2917624 |
| regulation of nitrogen compound metabolic process;cellular nitrogen compound metabolic process;nitrogen compound metabolic process | 2.272685 |
| regulation of nitrogen compound metabolic process;cellular nitrogen compound metabolic process;nitrogen compound metabolic process | 2.169787 |
| regulation of nitrogen compound metabolic process;cellular nitrogen compound metabolic process;nitrogen compound metabolic process | 2.0888922 |
| regulation of nitrogen compound metabolic process;cellular nitrogen compound metabolic process;nitrogen compound metabolic process | 2.0798106 |
| response to gibberellin stimulus;response to hormone stimulus | 7.460657 |
| response to gibberellin stimulus;response to hormone stimulus | 4.6277494 |
| response to gibberellin stimulus;response to hormone stimulus | 4.3567076 |
| response to gibberellin stimulus;response to hormone stimulus | 2.4714015 |
| response to hormone stimulus | 2.3990421 |
| response to hormone stimulus;regulation of nitrogen compound metabolic process;cellular nitrogen compound metabolic process;nitrogen compound metabolic process | 3.8964326 |
| response to hormone stimulus;regulation of nitrogen compound metabolic process;cellular nitrogen compound metabolic process;nitrogen compound metabolic process | 3.6240306 |
| response to hormone stimulus;response to abscisic acid stimulus | 7.676196 |
| response to hormone stimulus;response to abscisic acid stimulus | 4.7178216 |
| response to hormone stimulus;response to abscisic acid stimulus | 4.3420777 |
| response to hormone stimulus;response to abscisic acid stimulus | 3.2553174 |
| response to jasmonic acid stimulus | 3.8632944 |
| response to salicylic acid stimulus;response to gibberellin stimulus;response to ethylene stimulus;response to jasmonic acid stimulus;response to hormone stimulus;response to abscisic acid stimulus;regulation of nitrogen compound metabolic process;cellular nitrogen compound metabolic process;nitrogen compound metabolic process | 2.790834 |
| root epidermal cell differentiation;root morphogenesis;root system development;root development | 2.028872 |
| transferase activity, transferring acyl groups other than amino-acyl groups | 2.60415 |
| transferase activity, transferring acyl groups other than amino-acyl groups | 2.5963511 |
| transferase activity, transferring phosphorus-containing groups | 3.2462132 |
| transferase activity, transferring phosphorus-containing groups;phosphotransferase activity, phosphate group as acceptor | 2.5399575 |
| transferase activity, transferring phosphorus-containing groups;phosphotransferase activity, phosphate group as acceptor | 2.5047672 |
| transferase activity, transferring phosphorus-containing groups;phosphotransferase activity, phosphate group as acceptor | 2.035795 |
| VegetalPart | 7.4933214 |
| VegetalPart | 6.991514 |
| VegetalPart | 6.148426 |
| VegetalPart | 5.201164 |
| VegetalPart | 2.5605547 |
| VegetalPart | 2.231429 |
| VegetalPart | 2.1181002 |

**Table S2.2**

***Functionally characterized* *upregulated genes (p<0.05) for the roots of wheat plants treated with HA after 72 h from the onset of the treatment.***

| ***Description*** | **Fold Change** |
| --- | --- |
|  |  |
| aminoglycan catabolic process;aminoglycan metabolic process;nitrogen compound metabolic process | 2.8669534 |
| Azote | 17.478235 |
| Azote | 16.0345 |
| Azote | 11.557205 |
| Azote | 4.4609275 |
| Azote | 2.140116 |
| gibberellic acid mediated signaling pathway;response to gibberellin stimulus;cellular response to gibberellin stimulus;hormone-mediated signaling pathway;cellular response to hormone stimulus;gibberellin mediated signaling pathway;response to hormone stimulus | 2.50907 |
| Hormone | 3.2028515 |
| Hormone | 3.07715 |
| hormone-mediated signaling pathway;cellular response to hormone stimulus;response to hormone stimulus | 2.1070778 |
| ligase activity, forming carbon-nitrogen bonds;acid-amino acid ligase activity | 2.2860286 |
| Phosphore | 4.9351892 |
| Phosphore | 2.4807415 |
| Phosphore | 2.142152 |
| Phosphore | 2.0523865 |
| Phosphore | 2.0207498 |
| phosphorylation;phosphate metabolic process;phosphorus metabolic process;transferase activity, transferring phosphorus-containing groups;protein amino acid phosphorylation;phosphotransferase activity, alcohol group as acceptor | 2.1436477 |
| Photosynthesis | 3.1773236 |
| Photosynthesis;chloroplast thylakoid;photosynthesis, light reaction;chloroplast thylakoid membrane;chloroplast part;photosynthesis;chloroplast | 2.619848 |
| pyrophosphatase activity;nucleoside-triphosphatase activity;ligase activity, forming carbon-nitrogen bonds;acid-amino acid ligase activity;hydrolase activity, acting on acid anhydrides, in phosphorus-containing anhydrides | 3.8827994 |
| regulation of nitrogen compound metabolic process;cellular nitrogen compound metabolic process;nitrogen compound metabolic process | 2.6120474 |
| regulation of nitrogen compound metabolic process;cellular nitrogen compound metabolic process;nitrogen compound metabolic process | 2.1540525 |
| regulation of nitrogen compound metabolic process;cellular nitrogen compound metabolic process;nitrogen compound metabolic process | 2.0600839 |
| transferase activity, transferring phosphorus-containing groups | 2.623963 |
| VegetalPart | 3.4044952 |
| VegetalPart | 3.2456543 |
| VegetalPart | 2.3361619 |
| VegetalPart | 2.2051344 |
| VegetalPart | 2.1761158 |
| VegetalPart | 2.0875454 |
| VegetalPart | 2.0459754 |
| VegetalPart;response to jasmonic acid stimulus | 3.0516655 |
| VegetalPart;response to jasmonic acid stimulus | 3.04378 |

**ANNEX 3. Effects of HA on iP and iPR concentration in plant tissues**

**
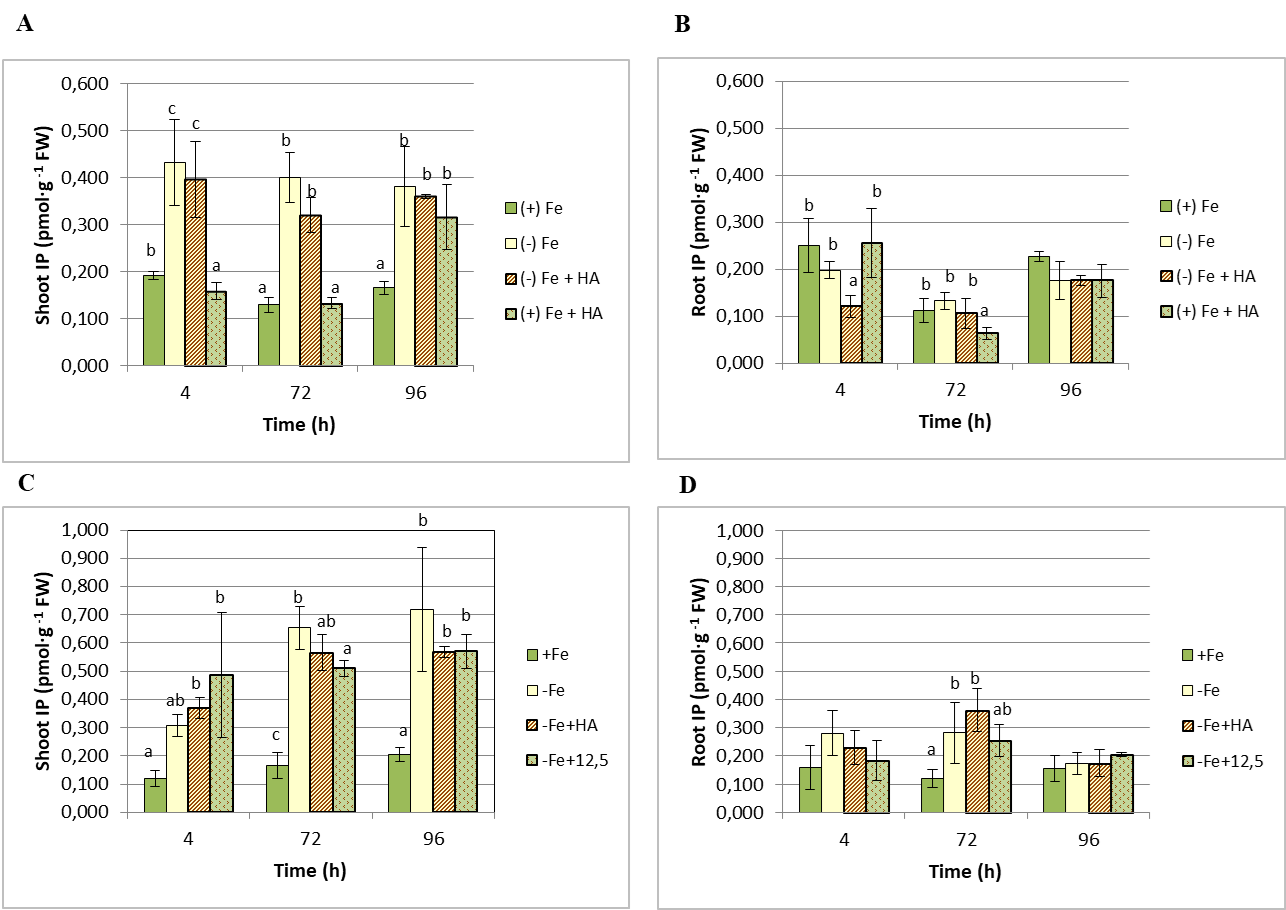
**

**Figure S3.1**. Impact of Fe deficiency and HA on iP concentration in plant. **(A)** Results in shoots for the first experiment. **(B)** Results in roots for the first experiment. (First experiment treatments: Plants with Fe ((+) Fe); Plants without Fe ((-) Fe); plants with Fe plus HA ((+) Fe + HA); plants without Fe plus HA ((-) Fe + HA)). **(C)** Results in shoots for the second experiment. **(D)** Results in roots for the second experiment. (Second experiment treatments: Plants with Fe ((+) Fe); Plants without Fe ((-) Fe); plants without Fe plus HA ((-) Fe + HA); plants plus 12.5 µM Fe ((-) Fe + 12.5)). Each data represents the average of three replicates with 23 plants per replicate. Bars represent standard deviation of the mean (SD). Different letters indicate significant differences between treatments for each time (ANOVA followed by a LSD Fischer test, P < 0.05).


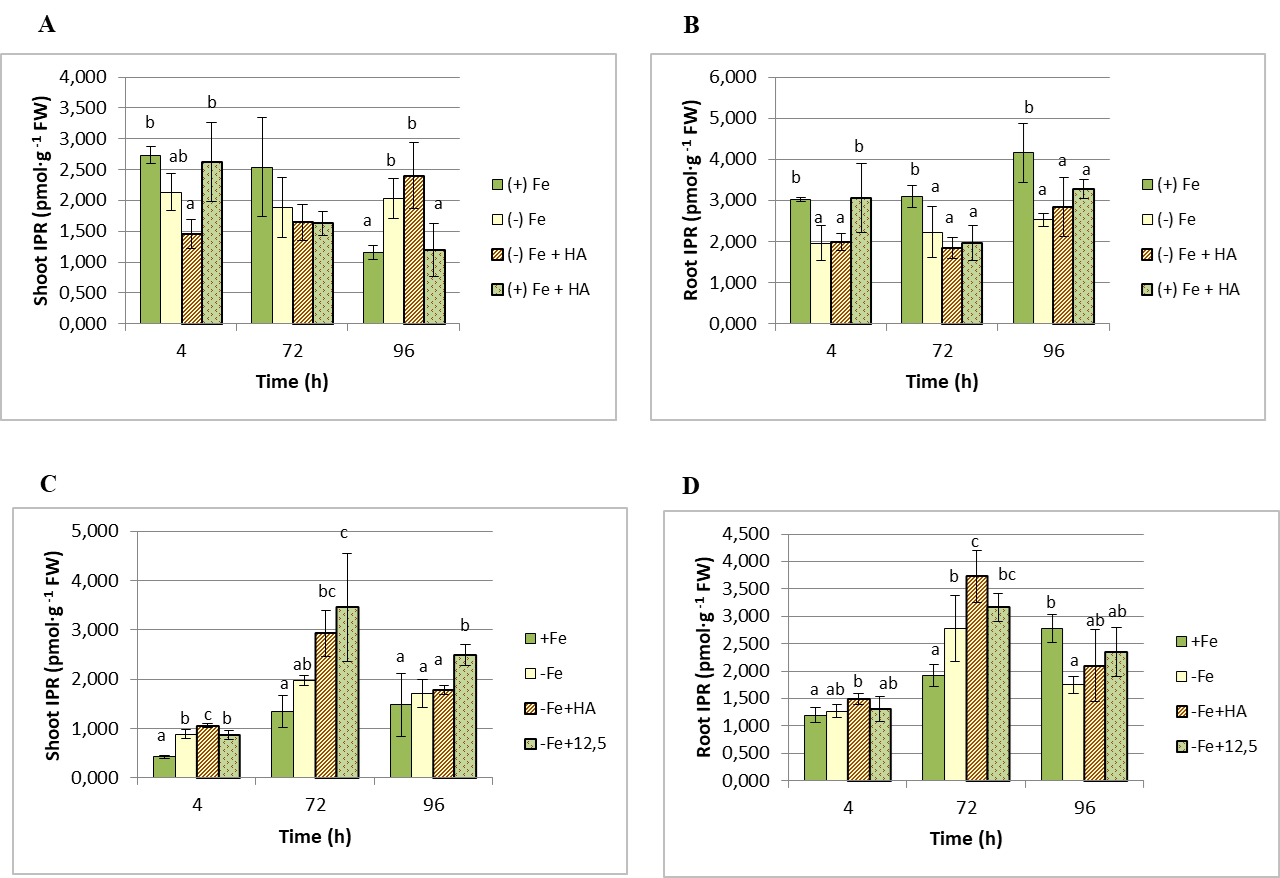


**Figure S3.2**. Impact of Fe deficiency and HA on iPR concentration in plant. **(A)** Results in shoots for the first experiment. **(B)** Results in roots for the first experiment. (First experiment treatments: Plants with Fe ((+) Fe); Plants without Fe ((-) Fe); plants with Fe plus HA ((+) Fe + HA); plants without Fe plus HA ((-) Fe + HA)). **(C)** Results in shoots for the second experiment. **(D)** Results in roots for the second experiment. (Second experiment treatments: Plants with Fe ((+) Fe); Plants without Fe ((-) Fe); plants without Fe plus HA ((-) Fe + HA); plants plus 12.5 µM Fe ((-) Fe + 12.5)). Each data represents the average of three replicates with 23 plants per replicate. Bars represent standard deviation of the mean (SD). Different letters indicate significant differences between treatments for each time (ANOVA followed by a LSD Fischer test, P < 0.05).
